# Resting neuroendocrine markers in relation to acute mental stress‐induced adrenergic reactivity profiles in adults: The SABPA study

**DOI:** 10.14814/phy2.70809

**Published:** 2026-03-16

**Authors:** Dewald Naudé, Wayne Smith, Roland von Känel, Annemarie Wentzel

**Affiliations:** ^1^ Hypertension in Africa Research Team (HART) North‐West University Potchefstroom South Africa; ^2^ South African Medical Research Council Unit for Hypertension and Cardiovascular Disease North‐West University Potchefstroom South Africa; ^3^ Department of Consultation‐Liaison Psychiatry and Psychosomatic Medicine, University Hospital, Zurich University of Zurich Zurich Switzerland

**Keywords:** acute mental stress, adrenocorticotropic hormone, alpha‐adrenergic response, beta‐adrenergic response, catecholamines, cortisol

## Abstract

Stress‐induced hemodynamic reactivity was categorized as predominant alpha (α)‐ and beta (β)‐adrenergic reactivity profiles. Within these profiles, we investigated resting neuroendocrine markers, their associations with hemodynamic reactivity, and odds of an α‐ or β‐adrenergic reactivity profile. We included 375 teachers (20–65 years) and recorded one‐minute beat‐to‐beat hemodynamic reactivity during Stroop‐Color‐Word‐Conflict‐test. We categorized α‐responders [lowest‐quartile ∆%CO, ∆%Cwk; *n* = 49], β‐responders [highest‐quartile ∆%CO, ∆%Cwk; *n* = 69], mixed‐α/β‐responders [remaining *n* = 257]. Baseline fasting serum adrenocorticotropic hormone (ACTH), cortisol, urinary norepinephrine‐to‐creatinine (u‐NE/Cr), and epinephrine‐to‐creatinine (u‐EPI/Cr) ratios were measured. Predominant α‐responders were older with greater hypertension prevalence than other responders. In α‐responders, u‐NE/Cr inversely, and ACTH and cortisol positively associated with ∆%CO and ∆%Cwk (all *p* ≤ 0.044). In β‐responders, u‐NE/Cr positively associated with ∆%CO, u‐EPI/Cr inversely with ∆%CO, and positively with ∆%Cwk (all *p* ≤ 0.045). Odds of an α‐profile were higher with u‐NE/Cr, ACTH, and cortisol in the highest‐quartile (all *p* ≤ 0.004). Odds of a β‐profile were higher with u‐NE/Cr in the highest‐quartile and ACTH and cortisol in the lowest‐quartile (all *p* ≤ 0.006). Predominant α‐responders exhibited higher u‐NE/Cr, ACTH, and cortisol, suggesting vascular risk through peripheral vasoconstriction. Predominant β‐responders showed higher u‐NE/Cr only, suggesting adaptive cardiac performance via catecholaminergic drive. These findings reveal distinct neuroendocrine underpinnings with implications for personalized acute stress cardiovascular phenotyping.

## Graphical abstract

A grapical summary of predominant alpha and beta repsonders' neuroendocrine profile in the SABAP cohort.

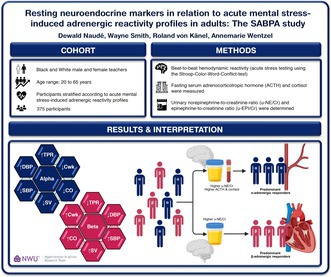

## 1 INTRODUCTION

Acute mental stress evokes hemodynamic reactivity responses characterized by stressor‐induced changes in heart rate (HR), stroke volume (SV), cardiac output (CO), blood pressure (BP), Windkessel arterial compliance (Cwk) and total peripheral resistance (TPR) (Williams, 1986). These hemodynamic responses are essential for the human body's adaptation to acute mental stress as they may aid in rapid adjustments in both cardiovascular and metabolic function to meet the increased demands imposed by stress (Ginty et al., 2017). Additionally, acute stress responses measured in a controlled clinical environment can be translated to the psychophysiological reactions observed in everyday stressful situations, particularly concerning cortisol (Steptoe, 1985). Within specific ethnic groups, previous studies have also linked individual components of the hemodynamic stress response (e.g., TPR and SV reactivity) to distinct indicators of cardiovascular disease (CVD) risk (Huisman et al., 2013; van Rooyen et al., 2002).

Acute mental stress‐induced hemodynamic reactivity response patterns are the result of a combination of alpha (α)‐ and/or beta (β)‐adrenergic receptor activation which is evoked by the autonomic nervous system (ANS) and the co‐activation of the physiologically interdependent sympatho‐adrenal‐medullary (SAM) and hypothalamic–pituitary–adrenal (HPA) axes (McEwen, 2007; Wadsworth et al., 2019). Evidence from the literature also suggests that neuroendocrine markers, including norepinephrine (NE), epinephrine (EPI), adrenocorticotropic hormone (ACTH), and cortisol are involved in the activation of these stress pathways (Godoy et al., 2018; Smith & Vale, 2006). While most studies have focused on individual hemodynamic reactivity parameters (Light et al., 1993; Light et al., 1994; Matthews et al., 2004), it may be more informative to investigate comprehensive adrenergic‐hemodynamic reactivity profiles. This approach offers a unique opportunity to more accurately characterize the hemodynamic stress response in its entirety.

We previously stratified a bi‐ethnic South African cohort into specific categories based on hemodynamic reactivity which reflect either a predominant α‐adrenergic reactivity profile, defined by the lowest quartile values of both cardiac output reactivity (∆%CO) and Windkessel arterial compliance reactivity (∆%Cwk), or a predominant β‐adrenergic reactivity profile, defined by the highest quartile values of both ∆%CO and ∆%Cwk (Wentzel et al., 2025). These adrenergic‐hemodynamic reactivity profiles may also inform CVD risk assessments, as previous work from our group showed that the predominant α‐ and β‐adrenergic reactivity profiles were associated with higher odds of several cardiovascular risk factors (Wentzel et al., 2025). However, also in our previous study, we did not include a mixed‐α/β‐adrenergic reactivity profile which would be advantageous to gain insights into the most prevalent hemodynamic reactivity response pattern in a study population. Whilst previous studies have acknowledged the complex interplay of the ANS and SAM‐ and HPA‐axes in the regulation of the acute stress response, and building on recent findings from our group, we aimed to investigate the specific manner in which classic neuroendocrine markers reflecting both SAM and HPA activity are associated with either a predominant α‐adrenergic, predominant β‐adrenergic, or mixed‐α/β‐adrenergic reactivity profile in humans.

We expected to observe unique associations between resting neuroendocrine markers and hemodynamic reactivity parameters within each adrenergic‐hemodynamic reactivity profile. In predominant α‐adrenergic responders, we hypothesized that NE will inversely associate with ∆%CO, which could reflect peripheral vasoconstrictive dominance (Motiejunaite et al., 2021) while ACTH and cortisol will positively associate with ∆%CO as cortisol may permissively potentiate NE functioning (Yang & Zhang, 2004). In predominant β‐adrenergic responders, positive associations of NE and EPI with ∆%CO and ∆%Cwk could be observed, which might signify enhanced cardiac performance and vascular compliance during stress (Goldstein, 2006). These unique relationships between neuroendocrine markers and adrenergic‐hemodynamic reactivity profiles are therefore important to investigate, as they enable us to gain a better understanding of the physiological foundation of each acute mental stress‐induced adrenergic reactivity profile. Therefore, in a South African cohort stratified by different adrenergic‐hemodynamic reactivity profiles, we aimed to (1) compare resting levels of neuroendocrine markers [urinary norepinephrine‐to‐creatinine ratio (u‐NE/Cr), urinary epinephrine‐to‐creatinine ratio (u‐EPI/Cr), serum ACTH and cortisol levels], (2) assess associations between neuroendocrine markers and hemodynamic reactivity parameters, and (3) determine the odds of neuroendocrine markers relating to a predominant adrenergic‐hemodynamic reactivity profile.

## 2 MATERIALS AND METHODS

### 2.1 Research design and participants

The study protocol of the Sympathetic activity and Ambulatory Blood Pressure in Africans (SABPA) prospective target population study is well‐described elsewhere and comprises a baseline and three‐year follow‐up phase (Malan et al., 2015). The baseline data collection phase took place from February to May during 2008 and 2009. For the current study, we included the baseline sample, consisting of urban‐dwelling Black and White South African male and female teachers (aged 20–65 years) from the Dr. Kenneth Kaunda education district in the North West province of South Africa (*N* = 409). These individuals were selected from a similar working environment, ensuring comparable socioeconomic status and educational background. Exclusion criteria of the original SABPA study included pregnant and/or lactating women, tympanic temperature >37.5°C, vaccination and blood donation within three months prior to data collection and individuals using psychotropic substances and/or α‐/β‐blockers. Participants with missing data for resting neuroendocrine markers (u‐NE/Cr, u‐EPI/Cr, serum ACTH, and cortisol) and hemodynamic reactivity parameters (∆%SBP, ∆%DBP, ∆%TPR, ∆%HR, ∆%SV, ∆%CO, ∆%Cwk) were additionally excluded (*n* = 34). The final study sample (*N* = 375) was grouped into three categories according to distinct adrenergic‐hemodynamic reactivity profiles as previously defined (Wentzel et al., 2025) (please also refer to Section 2.8).

### 2.2 Ethical considerations

The SABPA study complied with the Declaration of Helsinki of 1975 (revised 2004) for investigations involving human participants and was approved by the Health Research Ethics Committee of North‐West University (NWU‐00036‐07‐A6). All procedures, benefits and possible risks related to the study were explained to the participants in their preferred home language and their written informed consent was obtained before participation. The current investigation also adhered to the updated South African Department of Health's guidelines on Ethics in Health Research in human participants (2024).

### 2.3 General procedure of data collection

Data collection took place over a 2‐day period. Early on the morning of the first day, participants were fitted with an ambulatory blood pressure monitor (ABPM) device at their respective schools. At approximately 16:30, the participants were transported to the Metabolic Unit research facility of North‐West University for an overnight stay. Upon their arrival, they were familiarized with the experimental setup, after which they completed the questionnaires and received pre‐counseling for HIV/AIDS. At dinner time, the participants were provided with a standardized meal and instructed to fast overnight, with encouragement to go to bed by 22:00. At 05:45 the next morning, the ABPM device was removed and fasting urine samples collected over eight hours were obtained. Participants rotated between stations for various anthropometric and cardiovascular measurements, biological sampling, and acute mental stress testing procedures (using the Finometer and Stroop‐Color‐Word‐Conflict [Stroop‐CWC] test). After completing all measurements, they received post‐counseling for HIV/AIDS and immediate feedback on their health status. They were then thanked for their participation, given the opportunity to shower and have breakfast, and subsequently transported back to their respective schools.

### 2.4 Questionnaires and general demographics

After all study procedures were explained, participants completed the questionnaires in a private clinical research environment within the Metabolic Unit research facility at North‐West University. Before receiving a standardized dinner, they provided demographic and general health information, including age, sex, ethnicity, family history, lifestyle factors (such as self‐reported smoking and alcohol use), medication usage, and medical history.

### 2.5 Anthropometric measurements

All anthropometric measurements were taken by registered level II anthropometrists. Body height was determined to the nearest 0.1 cm using a stadiometer (Invicta stadiometer, IP 1465, Invicta Plastics Ltd., Leicester, UK) while body weight was measured to the nearest 0.1 kg with a standardized calibrated digital electronic scale (Precision Health Scale; A&D Company, Tokyo, Japan). Thereafter, body mass index (BMI) was calculated as body weight (in kg) divided by the square of body height (m^2^). Waist circumference (WC) was measured to the nearest 0.1 cm with a non‐extensible and flexible 7 mm‐wide metal anthropometric tape (Holtain, Croswell, Wales) at the midpoint between the lower costal rib and the iliac crest, perpendicular to the long axis of the trunk.

### 2.6 Ambulatory blood pressure measurements

The Cardiotens CE120® (Meditech, Budapest, Hungary), which was fitted with a suitable cuff size to the non‐dominant arm of each participant, was used to measure 24‐h ambulatory systolic (SBP) and diastolic (DBP) blood pressure. At approximately 08:00 on the first day of data collection, the validated ABPM device was fitted to the participants at their respective schools. The device was programmed to measure BP at 30‐min intervals during the day (08:00–22:00) and every hour during nighttime (22:00–06:00). The mean successful inflation rate over the 24‐h period was 74.1 ± 10.3% (Schutte et al., 2015). The 24‐h mean arterial pressure (MAP) was calculated as 23DBP+13SBP. Participants continued with their normal daily activities and recorded any abnormalities such as headache or nausea on their diary cards. The ABPM device was removed from the participants at 05:45 on the second day of data collection. The data was subsequently analyzed by means of the CardioVisions 1.19 Personal Edition Software (Meditech®). The current investigation defined hypertensive status as 24‐h ABPM SBP ≥130 mmHg and/or 24‐h ABPM DBP ≥80 mmHg and/or being on anti‐hypertensive medication, including angiotensin‐converting enzyme inhibitors, angiotensin II antagonists, thiazides/diuretics, and calcium‐channel blockers. This definition is derived from the 2020 International Society of Hypertension global hypertension practice guidelines according to average 24‐h ABPM readings (Unger et al., 2020).

### 2.7 Cardiovascular reactivity

Fasting cardiovascular measurements were continuously and non‐invasively assessed throughout acute mental stress testing using the validated Finometer device (Finapres Medical Systems®, Amsterdam, The Netherlands) (Guelen et al., 2008; Schutte et al., 2003, 2004). A five‐minute recording of each participant's beat‐to‐beat BP was taken after the participants were lying in a resting semi‐recumbent position for 30 min. After the first two minutes of this recording, a return‐to‐flow systolic calibration was performed to provide an individual subject‐level adjustment of the finger arterial pressure with brachial artery pressure. The highest precision in cardiovascular measurements is achieved only after this calibration, ensuring the BP measurements meet the standards of the Association for the Advancement of Medical Instrumentation. An additional resting period of five to ten minutes was provided to allow resting BP values to stabilize, after which the acute mental stress task (Stroop‐CWC test) was administered for one minute.

The Stroop‐CWC test was used to assess acute mental stress‐induced cardiovascular and neuroendocrine reactivity responses (Stroop, 1935) in which physiological changes characteristic of sympathoadrenal activation were evoked (Tulen et al., 1989). This stressor has demonstrated reproducibility in cardiovascular reactivity over both a two‐hour (Freyschuss et al., 1988) and a one‐month (Fauvel et al., 1996) period. Furthermore, the Stroop‐CWC test assesses the degree of cognitive interference control by inducing a mental conflict between incongruent colors and the meaning of printed words (e.g., the word “GREEN” printed in purple). This one‐minute procedure was administered by an independent observer who maintained a neutral facial expression while correcting incorrect responses and encouraging faster reactions. To motivate participants to perform well during the Stroop‐CWC test, they received monetary incentives based on their performance.

The average of the last three minutes of the resting recordings and the average of the last 20–30 s of the stressor recordings were used in data analyses to establish true baseline and reactivity observations. The Beat‐Scope version 1.1a software package (Finapres Measurement Systems) was used to calculate an integrated age‐dependent aortic flow curve from the surface area beneath the pressure/volume curve. This calculation determined SV, CO, TPR, and Cwk of the small and large arteries. Maximum cardiovascular reactivity of each hemodynamic parameter was calculated by first subtracting the resting values from the plateau values obtained during stress application and then calculating the percentage change (∆%) of each parameter.

### 2.8 Population stratification

The hemodynamic pattern related to α‐adrenergic predominance during acute stress exposure reflects elevated TPR and DBP with lower CO and Cwk which promotes peripheral vasoconstriction for vascular control (Kasprowicz et al., 1990; Sherwood & Turner, 1995; van Rooyen et al., 2002; Wentzel et al., 2019). In contrast, the hemodynamic pattern associated with β‐adrenergic predominance is characterized by heightened CO via increased HR and SV, thus reflecting myocardial activation for active coping (Kasprowicz et al., 1990; Sherwood & Turner, 1995; van Rooyen et al., 2002; Wentzel et al., 2019). Given that the hallmark hemodynamic patterns related to predominant α‐ and β‐adrenergic reactivity responses have previously been identified, we applied a quartile‐based mode of stratification to produce clearly defined dichotomized groups, i.e., predominant α‐ and β‐adrenergic responder groups, which were previously published (Wentzel et al., 2025). The predominant α‐adrenergic reactivity profile was therefore defined by the lowest quartile values of both ∆%CO and ∆%Cwk (*n* = 49) while the predominant β‐adrenergic reactivity profile was defined by the highest quartile values of both ∆%CO and ∆%Cwk (*n* = 69). The remainder of the study population was defined as the mixed‐α/β‐adrenergic reactivity profile (*n* = 257). We used both ∆%CO and ∆%Cwk as primary classification variables for adrenergic reactivity profiles, via hemodynamic patterns, firstly due to their combined hemodynamic relevance. Cardiac output is determined as the product of HR and SV (Frank, 1895) and serves as a key determinant of SBP (Vest, 2019). Additionally, Windkessel compliance measures the artery's ability to expand or relax in response to cardiac contraction in large and small arteries (Brar, 2016). This reflects the classic two‐element Windkessel model which describes the hemodynamics of the arterial system in terms of Cwk and TPR (Frank, 1895, 1899) and Poiseuille's law (Westerhof et al., 2009). Secondly, by combining both of these classification variables (i.e., ∆%CO and ∆%Cwk), one minimizes overadjustment and collinearity, but also take the overall hemodynamic pattern related to either α‐ or β‐adrenergic predominance during acute stress exposure into account, especially where inherent differences in adrenergic stress responses are observed (i.e., α‐response: low CO/Cwk; high TPR versus β‐response: high CO/Cwk; low TPR).

### 2.9 Biological sampling and biochemical analyses

Urinary creatinine concentrations were measured from fasting urine samples collected over a period of eight hours. These urine samples were obtained from each participant early in the morning (05:45) of the second day of data collection (Malan et al., 2015). Although studies have reported the preferred use of urine samples collected over 24 h for catecholamine sampling, both eight‐hour and 12‐h urine collection periods have been shown to yield comparable results for these urinary catecholamines (Reuben et al., 2000). Therefore, acidified samples (Maclagan, 2012) from the eight‐hour urine collection were used to measure urinary norepinephrine and epinephrine and were stored at −80°C until analysis within one year of collection. For catecholamine sampling, urine samples should be collected in containers with a pH of <3.5 to 4 (i.e., acidified with HCl) to prevent the breakdown of catecholamines and the samples should either be kept on ice or refrigerated until aliquoted (Maclagan, 2012). Urinary creatinine was used for volume correction; therefore, we reported urinary norepinephrine‐to‐creatinine ratio (u‐NE/Cr in nmol/mmol (Malan et al., 2016)) and epinephrine‐to‐creatinine ratio (u‐EPI/Cr in nmol/mmol). Urinary creatinine concentrations were measured using a calorimetric method via the Cobas® Integra 400 plus (Roche, Basel, Switzerland), while urinary NE and EPI were analyzed using a 3‐Cat Urine ELISA Fast Track kit (LDN, Nordhorn, Germany) (Catalogue number: BA E‐6600R). In this study, catecholamines were measured from acidified urine samples rather than blood samples due to (1) the short plasma half‐life of catecholamines (30 s to two minutes) (Peaston & Weinkove, 2004), (2) the complex procedure of plasma catecholamine sampling (Wentzel et al., 2020), and (3) the inability to use serum samples as catecholamines are stored in platelets and may be released during the clotting process (Maclagan, 2012).

Resting blood samples were collected by a trained SANC registered research nurse. Resting (pre‐stress) serum ACTH and cortisol samples were analyzed using the e411 (Roche, Basel, Switzerland) with the electrochemiluminescence immunoassay method. Fasting blood glucose samples were collected in sodium fluoride tubes and analyzed using a timed‐end‐point method on the UniCel DXC 800 (Beckman & Coulter, Germany). Serum insulin was analyzed with the electrochemiluminescence immunoassay method using the Elecsys 2010 (Roche, Basel, Switzerland). Thereafter, the homeostatic model assessment for insulin resistance (HOMA‐IR) was calculated (Matthews et al., 1985). Additionally, using a turbidimetric inhibition immunoassay method, glycated hemoglobin (HbA1c), in ethylenediaminetetraacetic acid (EDTA) whole blood samples, was analyzed on the Cobas® Integra 400 plus (Roche, Basel, Switzerland). Abnormal glucose tolerance (Abnl‐GT) is a composite term which includes both prediabetes and diabetes (Ishimwe et al., 2021). The current investigation defined Abnl‐GT as HbA1c ≥5.7% and/or fasting plasma glucose ≥5.6 mmol/L and/or being on anti‐diabetic medication (which included oral medication or using insulin for diabetes) according to the diagnostic criteria of the American Diabetes Association for prediabetes and diabetes (American Diabetes Association, 2019).

Serum total cholesterol, high‐density lipoprotein cholesterol (HDL‐C), and triglycerides were analyzed using a timed‐end‐point method on the UniCel DXC 800 (Beckman and Coulter, Germany). Low‐density lipoprotein cholesterol (LDL‐C) was calculated using the Friedewald formula (Fredrickson et al., 1972). The cholesterol‐to‐HDL‐C ratio (Chol/HDL‐C) was calculated by dividing total cholesterol by HDL‐C. Ultra‐high sensitivity serum C‐reactive protein (CRP) was analyzed using a turbidimetric method on the UniCel DXC 800 (Beckman & Coulter, Germany). Serum tumor necrosis factor‐alpha (TNF‐α) and plasma interleukin‐6 (IL‐6) values were derived from Quantikine High‐Sensitivity Human TNF‐α and IL‐6 enzyme‐linked immunosorbent assays (HS ELISA; R&D Systems, Minneapolis, MN USA) (Catalogue numbers HSTA00D and HS600C), respectively. Serum creatinine concentrations were measured using an enzymatic calorimetric method via the Cobas® Integra 400 plus (Roche, Basel, Switzerland) and were used for the calculation of the estimated glomerular filtration rate (eGFR) according to the 2021 Chronic Kidney Disease Epidemiology Collaboration (CKD‐EPI) Creatinine, Age and Sex equation (Inker et al., 2021).

### 2.10 Statistical analyses

Statistical analyses were performed using IBM® SPSS® Statistics version 29 software (IBM Corporation; Armonk, New York, USA). GraphPad Prism version 5.03 (GraphPad Software Inc., CA, USA) was used for the graphical illustration of data. Power analyses were performed for the larger SABPA study, and it was established that a sample size ranging from 50 to 416 would be sufficient to detect biological differences with a statistical power of 0.8 and significance level of 0.05 (Malan et al., 2015).

**FIGURE 1:**
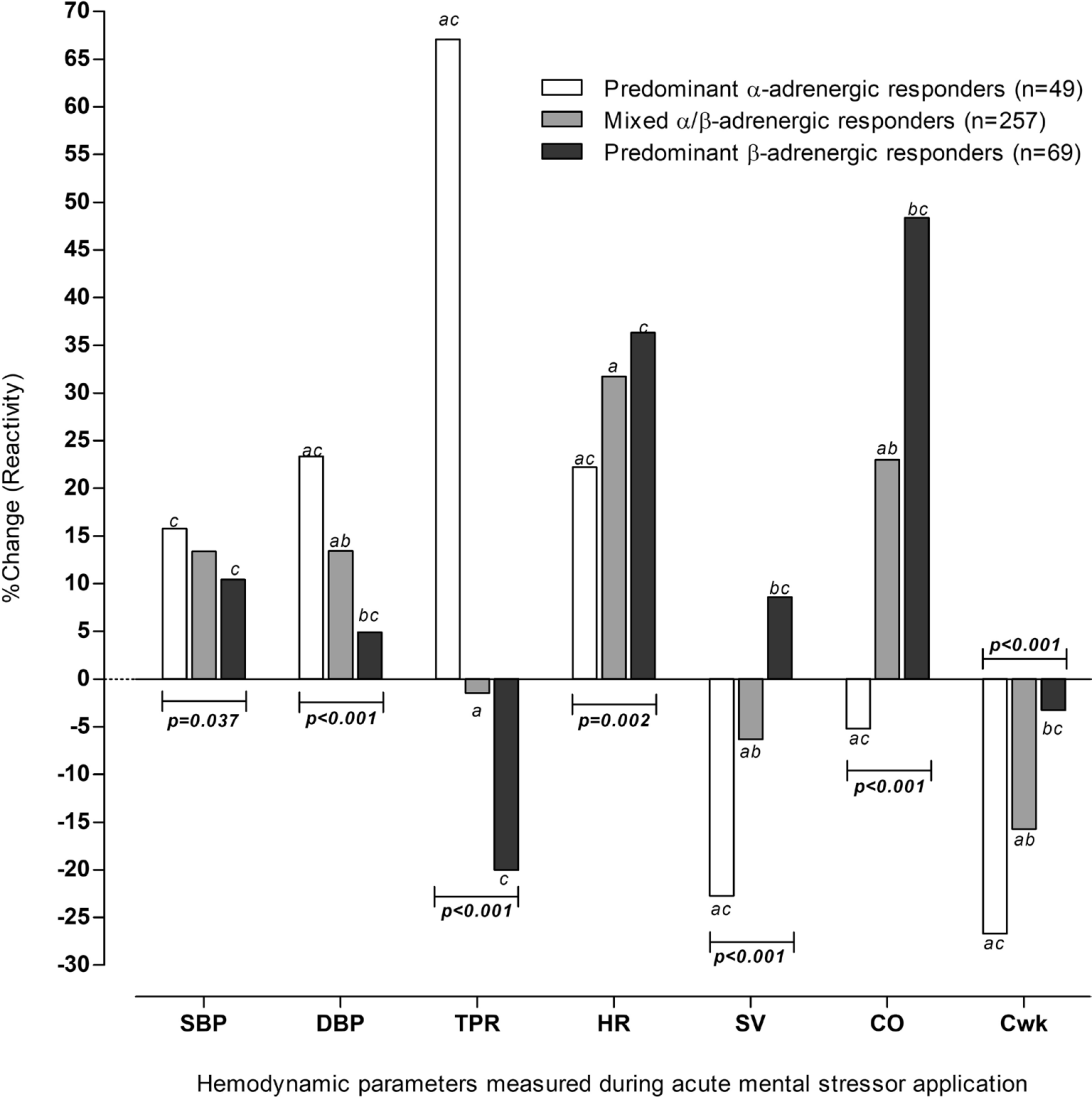
Comparison of hemodynamic reactivity parameters between acute mental stress‐induced adrenergic‐hemodynamic reactivity profiles, independent of age, sex, ethnicity, waist circumference, hypertensive status, abnormal glucose tolerance, self‐reported alcohol use, and self‐reported smoking. Symbols denote significant differences between ^a^predominant α‐adrenergic and mixed‐α/β‐adrenergic responders, ^b^predominant β‐adrenergic and mixed‐α/β‐adrenergic responders, and ^c^predominant α‐adrenergic and predominant β‐adrenergic responders. Bold values denote statistical significance (*p‐trend* <0.050). Where: CO, cardiac output; Cwk, Windkessel arterial compliance; DBP, diastolic blood pressure; HR, heart rate; SBP, systolic blood pressure; SV, stroke volume; TPR, total peripheral resistance.

All variables were assessed for normality by visual inspection of QQ‐plots and non‐Gaussian variables (ACTH, cortisol, u‐NE/Cr, u‐EPI/Cr, HbA1c, glucose, insulin, HOMA‐IR, IL‐6, TNF‐α, triglycerides, Chol/HDL‐C) were logarithmically transformed to the natural logarithm. Of note, hemodynamic reactivity parameters (i.e., ∆%SBP, ∆%DBP, ∆%TPR. ∆%SV, ∆%HR, ∆%CO, ∆%Cwk) were not logarithmically transformed as these parameters represent the percentage change in hemodynamic variables from baseline in response to acute stress application. Percentage changes are already normalized relative to baseline values, reducing the need for further transformation. Adjusted comparisons of all continuous variables between adrenergic‐hemodynamic reactivity profiles were performed using analyses of covariance (ANCOVA), with age, sex, and ethnicity as confounders. For hemodynamic reactivity parameters (∆%SBP, ∆%DBP, ∆%TPR, ∆%HR, ∆%SV, ∆%CO, and ∆%Cwk) presented in Figure 1, additional adjustments were made for WC, hypertensive status, Abnl‐GT, self‐reported alcohol use and self‐reported smoking, to ensure comparisons were independent of traditional confounders. Categorical variables were compared using Chi‐square tests. A two‐tailed significance level of *p* < 0.050 was considered statistically significant.

The most physiologically justified and statistically relevant confounders for both binomial logistic regression analyses (odds ratios) and backward stepwise multivariate regression analyses were chosen based on exploratory Spearman rank correlations between hemodynamic reactivity parameters (∆%CO and ∆%Cwk) as dependent variables and various other markers (age, sex, ethnicity, u‐NE/Cr, u‐EPI/Cr, ACTH, cortisol, BMI, WC, 24‐h ABPM BP, hypertensive status, glucose metabolism markers, lipids, inflammatory markers, eGFR, self‐reported smoking, self‐reported alcohol use) as independent variables (Table S1; Table S2–Supplementary material). The most significant confounders included age, sex, ethnicity, WC, hypertensive status, Abnl‐GT, self‐reported alcohol use, and self‐reported smoking. Due to the small sample size per group, no additional adjustments were made to avoid overadjustment and statistical artifacts.

Backward stepwise multivariate regression analyses were used to identify independent associations between neuroendocrine markers (u‐NE/Cr, u‐EPI/Cr, ACTH, cortisol) and hemodynamic reactivity parameters (∆%SV, ∆%CO, ∆%TPR, and ∆%Cwk) within each adrenergic reactivity profile. For multivariate regression analyses exclusively, hypertensive status was replaced with 24‐h ABPM MAP while additional confounders were included in the models of each profile due to their strong correlations observed with the hemodynamic reactivity parameters (∆%CO and ∆%Cwk). The eGFR was added to the predominant α‐adrenergic responder group models while CRP and Chol/HDL‐C were added to the mixed‐α/β‐adrenergic responder group models and IL‐6 to the predominant β‐adrenergic responder group models. In our regression analyses, we did not apply post‐hoc corrections for multiple hypothesis testing, as our study design was physiologically motivated and hypothesis‐generating. Each neuroendocrine marker and hemodynamic reactivity parameter was included in separate regression models to prevent our models becoming conflated with markers that are physiologically interrelated, and as the overall aim was to identify individual associations, separate models were therefore constructed.

Additionally, multivariate‐adjusted binomial logistic regression analyses were used to determine the odds of neuroendocrine markers (u‐NE/Cr, u‐EPI/Cr, ACTH, and cortisol) relating to a predominant adrenergic‐hemodynamic reactivity profile, that is, either the predominant α‐adrenergic or predominant β‐adrenergic reactivity profile.

## 3 RESULTS

### 3.1 Basic characteristics of participants

Baseline characteristics of the sample (*N* = 375) stratified according to the three acute mental stress‐induced adrenergic‐hemodynamic reactivity profiles are shown in Table 1. The sample comprised 49 predominant α‐adrenergic responders, 257 mixed‐α/β‐adrenergic responders, and 69 predominant β‐adrenergic responders. Predominant α‐adrenergic responders were mostly older, of Black ethnicity, had higher 24‐h ABPM MAP values, and greater prevalence of hypertension and Abnl‐GT compared to predominant β‐adrenergic and mixed‐α/β‐adrenergic responders (all *p* ≤ 0.029). There were no significant differences in u‐NE/Cr, u‐EPI/Cr, ACTH, and cortisol levels between the adrenergic‐hemodynamic reactivity profiles.

**TABLE 1:** Baseline characteristics of the study population stratified according to acute mental stress‐induced adrenergic‐hemodynamic reactivity profiles (*N* = 375).

	α‐Adrenergic Reactivity profile (*n* = 49)	Mixed‐α/β‐adrenergic Reactivity profile (*n* = 257)	β‐Adrenergic Reactivity profile (*n* = 69)	*p*
*Unadjusted analyses*				
Demographic and lifestyle information				
Age, years^†^	49.1 ± 7.60^ac^	44.7 ± 9.45^ab^	41.2 ± 11.0^bc^	**<0.001**
Sex, male, *n* (%)	23 (46.9)	133 (51.8)	29 (42.0)	0.34
Ethnicity, Black, *n* (%)	31 (63.3)^c^	123 (47.9)	17 (24.6)^c^	**<0.001**
Self‐reported smoking, yes, *n* (%)	8 (16.3)	35 (13.6)	13 (18.8)	0.53
Self‐reported alcohol use, yes, *n* (%)	14 (28.6)	101 (39.5)	27 (39.1)	0.35
Medical history				
Stroke, yes, *n* (%)	0 (0.0)	1 (0.4)	0 (0.0)	0.79
Myocardial infarction, yes, *n* (%)	0 (0.0)	2 (0.8)	1 (1.4)	0.68
Atrial fibrillation, yes, *n* (%)	2 (4.1)	9 (3.5)	5 (7.2)	0.39
Kidney disease, yes, *n* (%)	0 (0.0)	3 (1.2)^b^	5 (7.2)^b^	**0.004**
Diagnosed diabetes, yes, *n* (%)	2 (4.1)	9 (3.5)	0 (0.0)	0.27
Medication usage				
ACE inhibitors, yes, *n* (%)	5 (21.7)	16 (6.2)	2 (2.9)	0.26
Angiotensin II antagonists, yes, *n* (%)	1 (2.0)	2 (0.8)	1 (1.4)	0.69
Thiazides/Diuretics, yes, *n* (%)	6 (12.2)	22 (8.6)	1 (1.4)	0.065
Calcium‐channel blockers, yes, *n* (%)	3 (6.1)	11 (4.3)	1 (1.4)	0.41
Oral medication for diabetes, yes, *n (%)*	7 (14.3)	16 (6.2)	3 (4.3)	0.081
Using insulin for diabetes, yes, *n* (%)	3 (6.1)	4 (1.6)	2 (2.9)	0.15
Anti‐inflammatory medication, yes, *n* (%)	8 (16.3)^a^	12 (4.7)^a^	4 (5.8)	**0.009**
Adjusted analyses*				
Body composition				
Body mass index, kg/m^2^	28.4 (26.5;30.3)	29.6 (28.8;30.4)^b^	27.2 (25.6;28.8)^b^	**0.028**
Waist circumference, cm	92.7 (88.3;97.1)	94.8 (92.9;96.7)	90.2 (86.6;94.0)	0.088
Neuroendocrine markers				
Adrenocorticotropic hormone, pg/mL	15.7 (13.4;18.3)	15.7 (14.6;16.8)	15.2 (13.3;17.4)	0.93
Cortisol, nmol/L	299 (259;344)	349 (328;371)	324 (287;367)	0.11
u‐NE/Cr, nmol/mmol	19.4 (15.4;24.4)	19.7 (17.9;21.8)	17.4 (14.3;21.2)	0.55
u‐EPI/Cr, nmol/mmol	2.41 (1.91;3.04)	2.77 (2.50;3.06)	2.58 (2.11;3.13)	0.50
24‐h ambulatory blood pressure				
Systolic blood pressure, mmHg	133 (129;137)^c^	128 (127;130)	125 (121;128)^c^	**0.006**
Diastolic blood pressure, mmHg	82 (79;84)	80 (79;81)	78 (76;80)	0.10
Mean arterial pressure, mmHg	99 (96;102)^c^	96 (95;97)	94 (91;96)^c^	**0.029**
Hypertensive status, yes, *n* (%)	38 (77.6)^ac^	143 (55.6)^ab^	23 (33.3)^bc^	**<0.001**
Resting Finometer measurements				
Systolic blood pressure, mmHg	144 (139;148)^ac^	137 (135;139)^a^	136 (132;140)^c^	**0.015**
Diastolic blood pressure, mmHg	78 (75;80)	79 (78;80)	80 (77;82)	0.56
Total peripheral resistance, mmHg/mL/s	0.87 (0.75;1.00)^c^	0.99 (0.94;1.05)^b^	1.21 (1.10;1.31)^bc^	**<0.001**
Heart rate, bpm	70 (66;73)	67 (66;69)	66 (63;69)	0.23
Stroke volume, mL	114 (107;122)^ac^	99.9 (96.7;103)^a^	91.3 (85.0;97.5)^c^	**<0.001**
Cardiac output, L/min	7.72 (7.18;8.26)^ac^	6.64 (6.41; 6.88)^ab^	5.98 (5.53;6.43)^bc^	**<0.001**
Cwk, mL/mmHg	2.02 (1.91;2.13)	2.02 (1.97;2.07)	1.90 (1.81;2.00)	0.095
Biochemical analyses				
HbA1c, %	5.87 (5.67;6.07)	5.71 (5.63;5.80)	5.61 (5.45;5.78)	0.17
Glucose, mmol/L	5.71 (5.40;6.05)	5.60 (5.46; 5.74)	5.35 (5.10;5.61)	0.17
Insulin, μU/mL	11.5 (9.77;13.6)	11.2 (10.4;12.0)	9.86 (8.59;11.3)	0.24
HOMA‐IR	2.92 (2.42;3.52)	2.78 (2.57;3.02)	2.34 (2.00;2.74)	0.13
Abnormal glucose tolerance, yes, *n* (%)	35 (71.4)^c^	166 (64.6)	25 (36.2)^c^	**<0.001**
C‐reactive protein, mg/L	3.36 (2.53;4.47)	2.91 (2.58;3.29)	2.64 (2.08;3.36)	0.46
Interleukin‐6, pg/mL	1.04 (0.95;1.13)	1.01 (0.97;1.04)	1.00 (0.93;1.08)	0.80
Tumor necrosis factor‐alpha, pg/mL	1.34 (0.89;2.03)	1.16 (0.97;1.39)	0.97 (0.69;1.38)	0.50
Total cholesterol, mmol/L	5.06 (4.70;5.41)	5.15 (4.99;5.30)	5.26 (4.96;5.56)	0.70
HDL‐cholesterol, mmol/L	1.13 (1.03;1.23)	1.16 (1.12;1.20)	1.22 (1.14;1.31)	0.29
LDL‐cholesterol, mmol/L	3.64 (3.33;3.96)	3.73 (3.59;3.86)	3.78 (3.51;4.04)	0.82
Triglycerides, mmol/L	1.22 (1.05;1.43)	1.09 (1.02;1.02)	0.99 (0.87;1.12)	0.12
Cholesterol‐to‐HDL ratio	4.58 (4.18;5.03)	4.55 (4.37;4.73)	4.29 (3.97;4.64)	0.41
eGFR, mL/min/1.73 m^2^	99.1 (95.0;103)	99.6 (97.9;101)	97.7 (94.3;101)	0.63

*Note*: Values are expressed as arithmetic mean ± standard deviation, arithmetic mean (95% confidence intervals), geometric mean (lowest quartile; upper quartile) or frequency and percentage of participants (*n*, *%*). Bold values denote *p* < 0.050. All *p*‐values were obtained with chi‐squared tests, ^†^Welch's analysis of variance and *analysis of covariance (adjustments applied for age, sex, and ethnicity). Hypertensive status was defined as 24‐h ambulatory blood pressure ≥130/80 mmHg and/or being on anti‐hypertensive medication. Abnormal glucose tolerance was defined as HbA1c ≥5.7% and/or fasting plasma glucose ≥5.6 mmol/L and/or being on anti‐diabetic medication.

Abbreviations: ACE, angiotensin converting enzyme; Cwk, Windkessel arterial compliance; eGFR, estimated glomerular filtration rate; HbA1c, glycated hemoglobin; HDL, high‐density lipoprotein; HOMA‐IR, homeostatic model assessment for insulin resistance; LDL, low‐density lipoprotein; u‐EPI/Cr, urinary epinephrine‐to‐creatinine ratio; u‐NE/Cr, urinary norepinephrine‐to‐creatinine ratio.

^a^ α‐adrenergic responders and mixed‐α/β‐adrenergic responders.

^b^ β‐adrenergic responders and mixed‐α/β‐adrenergic responders.

^c^ α‐adrenergic responders and β‐adrenergic responders were obtained with Games‐Howell and Bonferroni post‐hoc tests.

After adjustment for age, sex, and ethnicity only, predominant α‐adrenergic responders showed greater increases in ∆%DBP and ∆%TPR along with a smaller increase in ∆%HR and greater decreases in ∆%SV, ∆%CO and ∆%Cwk compared to predominant β‐adrenergic and mixed‐α/β‐adrenergic responders (all *p* < 0.001). However, with additional adjustment for WC, hypertensive status, Abnl‐GT, self‐reported alcohol use and self‐reported smoking, these differences remained statistically significant while also observing greater increases in ∆%SBP in predominant α‐adrenergic responders compared to predominant β‐adrenergic and mixed‐α/β‐adrenergic responders (all *p* ≤ 0.037) (Figure 1).

### 3.2 The relationship between resting neuroendocrine markers and hemodynamic reactivity parameters

We explored independent associations of resting neuroendocrine markers (u‐NE/Cr, u‐EPI/Cr, ACTH, and cortisol) with ∆%CO (Table 2), ∆%Cwk (Table 3), ∆%SV (Table S3–Supplementary material) and ∆%TPR (Table S4–Supplementary material) in each adrenergic‐hemodynamic reactivity profile. In predominant α‐adrenergic responders, u‐NE/Cr was positively associated with ∆%TPR (*p* = 0.029) and inversely with ∆%CO (*p* = 0.002), ∆%SV (*p* = 0.003), and ∆%Cwk (*p* = 0.029). In addition, u‐EPI/Cr was inversely associated with ∆%SV (*p* = 0.048). Furthermore, both ACTH and cortisol were positively associated with ∆%CO (all *p* ≤ 0.003), ∆%Cwk (all *p* ≤ 0.044) and ∆%TPR (all *p* ≤ 0.043) and inversely with ∆%SV (all *p* ≤ 0.008).

**TABLE 2:** Backward stepwise regression analyses of resting neuroendocrine markers and cardiac output reactivity (∆%CO) stratified according to acute mental stress‐induced adrenergic–hemodynamic reactivity profiles (*N* = 375).

	Predominant α‐adrenergic reactivity profile (*n* = 49)		Mixed‐α/β‐adrenergic reactivity profile (*n* = 257)		Predominant β‐adrenergic reactivity profile (*n* = 69)	
	ß (± 95% CI)	*p*	ß (± 95% CI)	*p*	ß (± 95% CI)	*p*
*Dependent variable: Cardiac output reactivity (∆%CO)*						
Adj *R* ^2^	0.25		0.13		0.35	
u‐NE/Cr	−0.78 (−1.08; −0.53)	**0.002**	NS	NS	2.54 (2.07; 2.91)	**<0.001**
Age	NS	NS	−0.47 (−0.73; −0.12)	**0.037**	NS	NS
Ethnicity	1.25 (−0.98; 2.05)	0.098	5.44 (−0.52; 11.4)	0.073	NS	NS
Waist circumference	−0.23 (−0.58; −0.03)	**0.017**	NS	NS	NS	NS
24‐h ABPM MAP	NS	NS	NS	NS	−2.12 (−2.69; −1.15)	**0.011**
Abnl‐GT	−3.68 (−4.24; −2.85)	**0.009**	NS	NS	2.72 (1.52; 3.21)	**<0.001**
Interleukin‐6	NS	NS	NS	NS	NS	NS
C‐reactive protein	0.85 (0.03; 1.63)	**0.038**	−2.96 (−5.64; −0.28)	**0.031**	NS	NS
Adj *R* ^2^	0.12		<0.010		0.11	
u‐EPI/Cr	NS	NS	3.50 (0.14; 6.88)	**0.047**	−7.15 (−19.5; −0.28)	**0.036**
Age	NS	NS	−0.41 (−0.71; −0.11)	0.072	NS	NS
Waist circumference	−0.33 (−0.70; −0.07)	**0.039**	NS	NS	NS	NS
24‐h ABPM MAP	NS	NS	NS	NS	−0.84 (−1.87; −0.28)	**0.048**
Abnl‐GT	NS	NS	NS	NS	NS	NS
Interleukin‐6	NS	NS	NS	NS	NS	NS
C‐reactive protein	NS	NS	−0.63 (−2.46; 1.89)	0.069	NS	NS
Adj *R* ^2^	0.32		0.11		0.23	
ACTH	2.34 (2.14; 3.08)	**<0.001**	NS	NS	NS	NS
Age	NS	NS	−0.23 (−0.48; −0.08)	**0.003**	NS	NS
Ethnicity	NS	NS	NS	NS	NS	NS
Waist circumference	−0.36 (−0.70; −0.01)	**0.042**	NS	NS	NS	NS
24‐h ABPM MAP	NS	NS	NS	NS	−1.23 (−1.95; −0.58)	**0.022**
Abnl‐GT	−3.12 (−4.21; −1.56)	**0.025**	NS	NS	1.12 (0.55; 1.42)	**0.001**
Interleukin‐6	NS	NS	NS	NS	NS	NS
C‐reactive protein	NS	NS	−0.26 (−2.32; −0.05)	0.083	NS	NS
Adj *R* ^2^	0.30		0.10		0.18	
Cortisol	4.58 (2.54; 8.98)	**0.003**	NS	NS	NS	NS
Age	NS	NS	−0.38 (−0.72; −0.02)	**0.031**	NS	NS
Ethnicity	NS	NS	NS	NS	NS	NS
24‐h ABPM MAP	NS	NS	NS	NS	−2.01 (−3.59; −1.02)	**0.008**
Abnl‐GT	−4.21 (−6.98; −1.64)	**0.013**	NS	NS	2.40 (2.03; 4.72)	**0.001**
Interleukin‐6	NS	NS	NS	NS	−1.38 (−2.05; −0.03)	**0.048**
C‐reactive protein	NS	NS	−2.67 (−4.25; −0.98)	0.097	NS	NS

*Note*: All models were adjusted for age, sex, ethnicity, waist circumference, 24‐h ambulatory mean arterial pressure (ABPM MAP), abnormal glucose tolerance (Abnl‐GT), self‐reported smoking, and self‐reported alcohol use. For models specific to α‐adrenergic responders, estimated glomerular filtration rate was added. For models specific to mixed‐α/β‐adrenergic responders, C‐reactive protein and cholesterol‐to‐HDL ratio were added. For models specific to β‐adrenergic responders, interleukin‐6 was added. Bold values denote statistical significance (*p* < 0.050).

Abbreviations: ACTH, adrenocorticotropic hormone; CI, confidence interval; NS, not significant; u‐EPI/Cr, urinary epinephrine‐to‐creatinine ratio; u‐NE/Cr, urinary norepinephrine‐to‐creatinine ratio.

**TABLE 3:** Backward stepwise regression analyses of resting neuroendocrine markers and Windkessel arterial compliance reactivity (∆%Cwk) stratified according to acute mental stress‐induced adrenergic‐hemodynamic reactivity profiles (*N* = 375).

	Predominant α‐adrenergic Reactivity profile (*n* = 49)		Mixed‐α/β‐adrenergic Reactivity profile (*n* = 257)		Predominant β‐adrenergic Reactivity profile (*n* = 69)	
	ß (± 95% CI)	*p*	ß (± 95% CI)	*p*	ß (± 95% CI)	*p*
Dependent variable: Windkessel arterial compliance reactivity (∆%Cwk)						
Adj *R* ^2^	0.24		<0.10		0.15	
u‐NE/Cr	−0.37 (−0.65; 0.09)	**0.029**	NS	NS	0.28 (0.01; 0.42)	0.051
Waist circumference	NS	NS	NS	NS	0.24 (0.037; 0.44)	**0.021**
Abnl‐GT	NS	NS	NS	NS	7.81 (0.86; 14.8)	**0.028**
Adj *R* ^2^	<0.10		<0.10		0.16	
u‐EPI/Cr	NS	NS	−0.98 (−1.54; 0.08)	0.15	0.31 (0.06; 0.64)	**0.045**
Waist circumference	NS	NS	NS	NS	0.24 (0.037; 0.44)	**0.021**
Abnl‐GT	NS	NS	NS	NS	7.81 (0.86; 14.8)	**0.028**
Adj *R* ^2^	0.27		<0.10		0.21	
ACTH	2.31 (1.11; 3.58)	**0.044**	NS	NS	NS	NS
Waist circumference	NS	NS	NS	NS	0.38 (0.06; 0.52)	**0.022**
Abnl‐GT	NS	NS	NS	NS	5.88 (1.45; 9.18)	**0.039**
Adj *R* ^2^	0.30		<0.10		0.10	
Cortisol	3.66 (0.21; 7.05)	**0.037**	NS	NS	NS	NS
Waist circumference	NS	NS	NS	NS	0.22 (0.05; 0.49)	**0.009**
Abnl‐GT	NS	NS	NS	NS	6.94 (2.68; 10.1)	**0.019**

*Note*: All models were adjusted for age, sex, ethnicity, waist circumference, 24‐h ambulatory mean arterial pressure (ABPM MAP), abnormal glucose tolerance (Abnl‐GT), self‐reported smoking, and self‐reported alcohol use. For models specific to α‐adrenergic responders, estimated glomerular filtration rate was added. For models specific to mixed‐α/β‐adrenergic responders, C‐reactive protein and cholesterol‐to‐HDL ratio were added. For models specific to β‐adrenergic responders, interleukin‐6 was added. Bold values denote statistical significance (*p* < 0.050).

Abbreviations: ACTH, adrenocorticotropic hormone; CI, confidence interval; NS, not significant; u‐EPI/Cr, urinary epinephrine‐to‐creatinine ratio; u‐NE/Cr, urinary norepinephrine‐to‐creatinine ratio.

In predominant β‐adrenergic responders, u‐NE/Cr was positively associated with ∆%CO (*p* < 0.001) and ∆%SV (*p* = 0.019) and inversely with ∆%TPR (*p* = 0.033). Additionally, u‐EPI/Cr was inversely associated with ∆%CO (*p* = 0.036), yet positively with ∆%Cwk (*p* = 0.045) and ∆%SV (*p* = 0.012). Cortisol was inversely associated with ∆%TPR (*p* = 0.018).

In mixed‐α/β‐adrenergic responders, u‐EPI/Cr was positively associated with ∆%CO (*p* = 0.047) and ∆%SV (*p* = 0.009) while u‐NE/Cr was positively associated with ∆%SV (*p* = 0.007). Additionally, ACTH associated inversely with ∆%SV (*p* = 0.029).

### 3.3 The odds of resting neuroendocrine markers relating to a specific adrenergic‐hemodynamic reactivity profile

**FIGURE 2:**
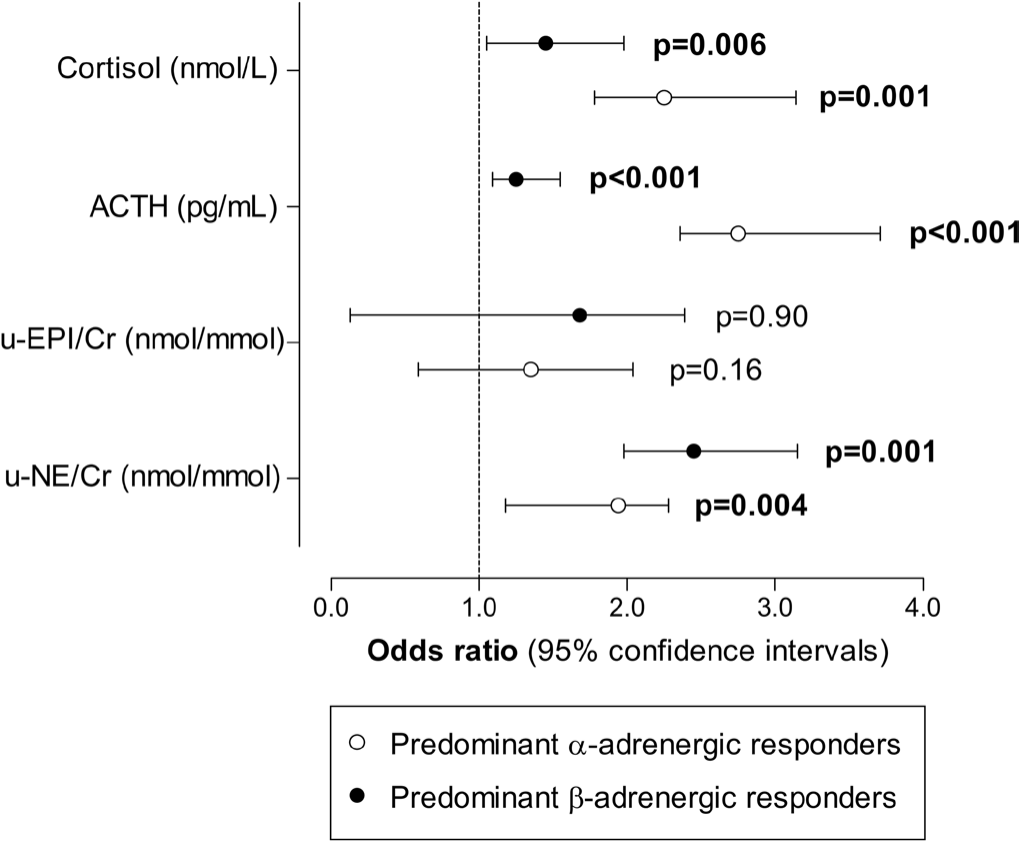
The odds of a predominant α‐adrenergic reactivity profile when u‐NE/Cr, u‐EPI/Cr, ACTH, and cortisol levels were within highest population quartile ranges (white dots). Black dots indicate the odds of a predominant β‐adrenergic reactivity profile when u‐NE/Cr and u‐EPI/Cr were within highest population quartile ranges and when ACTH and cortisol levels were within lowest‐population‐quartile ranges. Data in boldface denotes statistical significance. ACTH, adrenocorticotropic hormone; u‐EPI/Cr, urinary epinephrine‐to‐creatinine ratio; u‐NE/Cr, urinary norepinephrine‐to‐creatinine ratio.

We explored the odds of a predominant α‐adrenergic reactivity profile when u‐EPI/Cr, u‐NE/Cr, ACTH, and cortisol levels were in the highest quartile (Figure 2; white dots). However, for the predominant β‐adrenergic reactivity profile, linear regression analyses showed an inverse association with both ACTH and cortisol. Therefore, we explored the odds of a predominant β‐adrenergic reactivity profile when u‐NE/Cr and u‐EPI/Cr were in the highest quartile and ACTH and cortisol in the lowest quartile ranges (Figure 2; black dots). We found that the odds of a predominant α‐adrenergic profile were higher when u‐NE/Cr (OR = 1.94; 95% CI: 1.18, 2.28; *p* = 0.004), ACTH (OR = 2.75; 95% CI: 2.36, 3.71; *p* < 0.001), and cortisol (OR = 2.25; 95% CI: 1.78, 3.14; *p* = 0.001) levels were in the highest quartile. In contrast, the odds of a predominant β‐adrenergic reactivity profile were higher when u‐NE/Cr levels were in the highest quartile (OR = 2.45; 95% CI: 1.98, 3.15; *p* = 0.001) and when ACTH (OR = 1.25; 95% CI: 1.09, 1.55; *p* < 0.001) and cortisol (OR = 1.45; 95% CI: 1.05, 1.98; *p* = 0.006) levels were in the lowest quartile.

### 3.4 Sensitivity analyses

Participants using α‐ and/or β‐blockers were excluded from the original SABPA study. However, a subsequent data review revealed that six participants (n=6) were prescribed β‐blockers as anti‐hypertensive treatment, and were inadvertently included in the final dataset. To address this, we performed a sensitivity analysis by excluding these participants and repeating the multivariate regression analyses upon which we observed that the results remained unchanged.

## 4 DISCUSSION

The current study shows the distinct relationship between resting neuroendocrine markers (u‐NE/Cr, u‐EPI/Cr, ACTH, cortisol) and stress‐induced hemodynamic reactivity parameters (∆%SV, ∆%CO, ∆%Cwk, ∆%TPR) within specific acute adrenergic‐hemodynamic reactivity profiles. Although these neuroendocrine markers did not differ between adrenergic‐hemodynamic reactivity profiles, we found that the manner in which they were associated with hemodynamic reactivity parameters differed significantly within each reactivity profile, independent of a range of potentially confounding variables. Moreover, this study is the first to describe predominant α‐ and β‐adrenergic reactivity profiles based on distinct neuroendocrine signatures, which may offer insights into personalized treatment strategies tailored to the CVD risk associated with each reactivity profile.

### 4.1 The predominant α‐adrenergic reactivity profile

**FIGURE 3:**
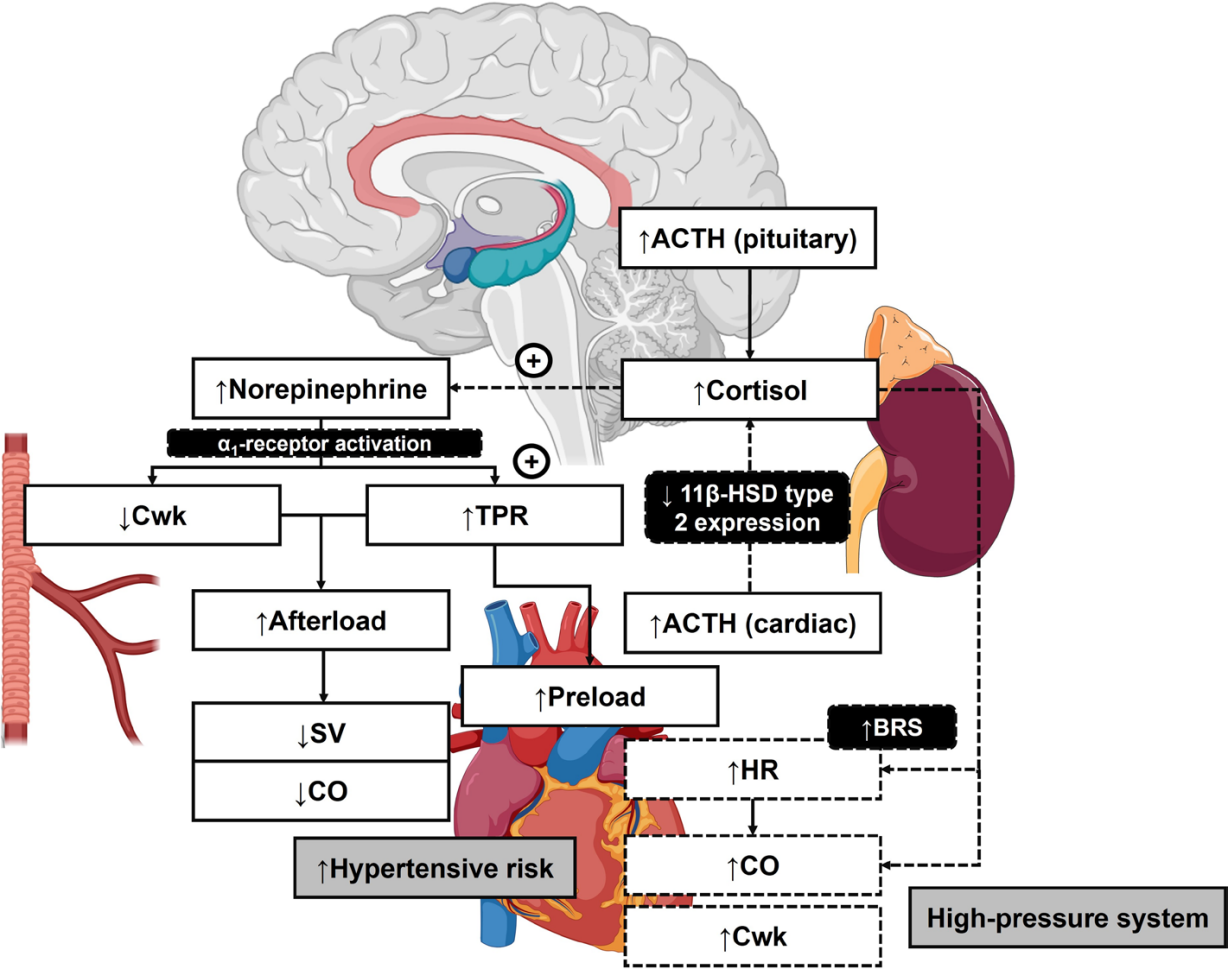
Hypothetical mechanisms which relate resting neuroendocrine markers to hemodynamic reactivity parameters in predominant α‐adrenergic responders. 11β‐HSD, 11β‐hydroxysteroid dehydrogenase; ACTH, adrenocorticotropic hormone; BRS, baroreceptor sensitivity; CO, cardiac output; Cwk, Windkessel arterial compliance; HR, heart rate; SV, stroke volume; TPR, total peripheral resistance. Figure compiled using elements from BioRender (https://apps.biorender.com) and SMART Servier® Medical Art (Creative Commons Attribution 3.0 Unported License, see https://smart.servier.com).

Microneurography provides a direct method to measure postganglionic sympathetic nerve activity (Grassi & Esler, 1999). However, its use in clinical practice is limited due to its invasive nature (Rahman et al., 2025). Therefore, we measured urinary catecholamines over a period of eight hours as a proxy marker for acute catecholamine release during sympathetic nervous system (SNS) outflow (Reuben et al., 2000) which may reflect cumulative catecholamine release during extended periods of stress exposure such as in everyday life (Bosker et al., 2012; Ward & Mefford, 1985). Therefore, it is possible that our findings, through urinary excretion of NE, may reflect every day, excessive peripheral SNS‐driven responses favoring enhanced vascular smooth muscle cell (VSMC) constriction (via vascular α_1_‐adrenergic receptor activation by NE) and hemodynamically translates to increased TPR and lower Cwk (MacGregor et al., 1974; Opie, 2004; Smiley et al., 1998) (see Figure 3). Higher TPR may increase cardiac afterload leading to decreased SV and CO, thus increasing cardiac preload during acute stress application (Katz et al., 2019; Vest, 2019). The observed increase in ∆%HR might represent an adaptive attempt to manage the stressor despite the greater peripheral vascular response. This could suggest a preference for NE binding to α‐adrenergic receptors in the periphery rather than its effect on the sinoatrial node in cardiac tissue (Toyoda et al., 2023). This pattern may also indicate reduced β‐adrenergic responsiveness in α‐adrenergic responders (Julius, 1994). However, further investigation is required to determine α‐ and β‐adrenergic receptor density and sensitivity within the context of predominant adrenergic‐hemodynamic reactivity profiles to verify this hypothesis. Additionally, the predominant vascular α‐adrenergic reactivity pattern, which is accompanied by higher 24‐h BP and greater hypertension prevalence, may further reflect a sustained high‐pressure system (Wentzel et al., 2025) which may increase an individual's predisposition to structural remodeling of the vasculature, as previously reported (Malan et al., 2010).

Our results further support cortisol's permissive effect on catecholamine functioning, via glucocorticoid receptors (Yang & Zhang, 2004), in which resting cortisol may potentiate norepinephrine's effects on the VSMCs of the peripheral blood vessels, thus further exacerbating α‐adrenergic‐mediated vasoconstriction (increased TPR). However, it is evident from cortisol's positive associations with both ∆%CO and ∆%Cwk that cortisol may also contribute to increased cardiac contractility via its positive inotropic effect (Whitworth et al., 2005). In addition, and independent of its permissive effect on NE functioning, resting cortisol may also rapidly increase baroreceptor sensitivity of heart rate control (Schulz et al., 2020). Therefore, we carefully suggest that the direct effects of cortisol may act as a compensatory mechanism to counterbalance the observed decrease in CO induced by NE‐mediated vasoconstriction. This may result in increased Cwk, despite the observed increase in TPR.

Cortisol secretion by the adrenal cortex is physiologically regulated by means of ACTH released by the anterior pituitary gland (Smith & Vale, 2006). The observed positive association between resting ACTH and ∆%CO in the predominant α‐adrenergic responder group may support previous findings which indicated that ACTH may also directly enhance CO and arterial pressure. Here, cortisol's effects on vascular tone may be potentiated by means of ACTH‐mediated decreases in gene expression and enzyme activity of 11β‐hydroxysteroid dehydrogenase type 2 in human aortic endothelial cells, thus preventing cortisol from being converted to its inactive metabolite, cortisone (Hatakeyama et al., 2000). Therefore, our findings are the first to confirm the resting state function of the HPA‐axis as reflected by a unique adrenergic‐hemodynamic reactivity profile in humans.

### 4.2 The predominant β‐adrenergic reactivity profile

**FIGURE 4:**
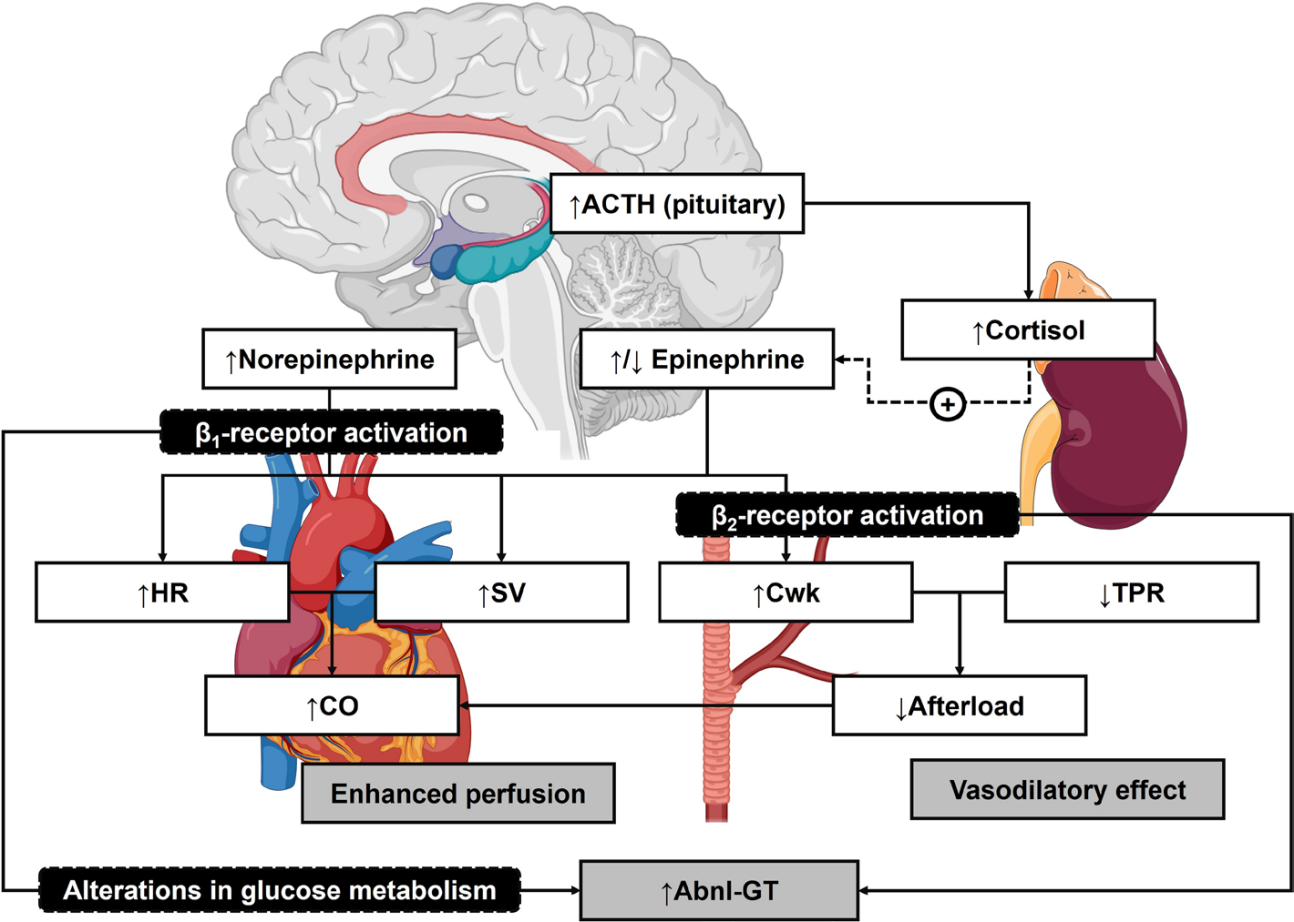
Hypothetical mechanisms which relate resting neuroendocrine markers to hemodynamic reactivity parameters in predominant β‐adrenergic responders. Abnl‐GT, abnormal glucose tolerance; ACTH, adrenocorticotropic hormone; CO, cardiac output; Cwk, Windkessel arterial compliance; HR, heart rate; SV, stroke volume; TPR, total peripheral resistance. Figure compiled using elements from BioRender (https://apps.biorender.com) and SMART Servier® Medical Art (Creative Commons Attribution 3.0 Unported License, see https://smart.servier.com).

In the predominant β‐adrenergic responder group, 24‐h BP and hypertension prevalence were lower than compared to the predominant α‐adrenergic responder group. It is important to note that although both β_1_‐ and β_2_‐adrenergic receptor subtypes are highly homologous and are both expressed in cardiac tissue, they both play distinguishable roles in the regulation of cardiac function (Opie, 2004). While both receptor subtypes physiologically have a similar affinity for EPI, the β_1_‐subtype has a tenfold higher affinity for NE than the β_2_‐subtype (Xu et al., 2021). It has also been reported that urinary NE and EPI are more sensitive indicators of circulating plasma NE and EPI, respectively, compared to catecholamine metabolites such as vanillylmandelic acid and 3‐methoxy‐4‐hydroxyphenylglycol (Moleman et al., 1992). Therefore, it is plausible that the positive associations observed between u‐NE/Cr with ∆%CO and ∆%SV may reflect an enhanced positive inotropic effect exerted by NE on cardiomyocytes via β_1_‐adrenergic receptor activation (see Figure 4). It may also be viable to explain the associations of u‐EPI/Cr with ∆%CO and ∆%Cwk within the context of heightened β‐adrenergic receptor sensitivity for EPI. Indeed, EPI (at high or low levels) may enhance cardiac contractility to increase the CO (via the β_1_‐subtype) but also elicit an enhanced vasodilatory effect which ultimately could reduce TPR and increase Cwk (via the β_2_‐subtype) (Motiejunaite et al., 2021).

Additionally, cortisol facilitates EPI synthesis (from NE) in the adrenal medulla by promoting the activity of phenylethanolamine N‐methyltransferase (Goldstein, 2006). Therefore, it is possible that with increased production of EPI (indirectly via the effects of cortisol), the overall vasodilatory effect, via arteriolar β_2_‐adrenergic receptor activation, is enhanced even further to reduce TPR and increase blood flow to peripheral organs. These findings suggest that the predominant β‐adrenergic reactivity profile reflects a central cardiac SNS‐driven response which may be advantageous in the short term, as supported by previous findings (Wentzel et al., 2019). Importantly, one must also consider that this hemodynamic reactivity response may become detrimental over time, as hyperperfusion of tissues could induce increased shear stress which may promote increased oxidative stress and endothelial dysfunction (Huang et al., 2013). However, as our study is cross‐sectional, we can only speculate on CVD mechanisms within each adrenergic‐hemodynamic reactivity profile.

Interestingly, Abnl‐GT and WC contributed significantly to the variance of our regression models for the predominant β‐adrenergic reactivity profile. Sustained β‐adrenergic receptor stimulation may also lead to alterations in glucose metabolism, and the development of insulin resistance (IR) may also become evident (Cipolletta et al., 2009). This suggests that Abnl‐GT, potentially driven by early‐stage IR, could pose a greater metabolic risk for predominant β‐adrenergic responders. However, further investigation is required as we did not investigate associations between hemodynamic reactivity parameters and metabolic markers, nor did we stratify participants according to different etiologies of Abnl‐GT.

### 4.3 The mixed‐α/β‐adrenergic reactivity profile

The mixed‐α/β‐adrenergic reactivity profile may result from combined activation of both α‐ and β‐adrenergic receptors. Based on the few associations observed in this group, we cautiously propose that this response pattern may in part reflect a coordinated hemodynamic response in which the SAM‐ and HPA‐axes effectively regulate cardiovascular performance during acute stress to accommodate the heightened demand for perfusion to the periphery (Rotenberg & McGrath, 2016), which could signify possible physiological adaptability in response to acute stress. Interestingly, this group showed the highest values for WC compared to predominant α‐ and β‐adrenergic responders. Additionally, WC contributed to the variance of the regression models of ACTH and cortisol with ∆%SV. This indicates that this responder group is not without CVD risk as other traditional cardiovascular risk factors may also contribute to the individual's CVD risk profile. As we did not investigate associations between neuroendocrine and adiposity markers, further exploration is warranted to determine whether interactions between HPA‐axis markers and for instance visceral adiposity could explain the CVD risk related to the mixed‐α/β‐adrenergic reactivity profile. The limited associations observed in this group warrant careful consideration and may, in part, also reflect methodological constraints, as this group encompasses a continuum of varying degrees of α‐ and β‐adrenergic receptor activation in which the observed heterogeneity in adrenergic predominance may obscure more specific associations in this group. Considering appropriate group sizes, future studies may conduct further sub‐group analyses in this responder group in order to clarify the associations observed.

### 4.4 Translational and clinical relevance

Addressing psychophysiological stress on an equal level with traditional cardiovascular risk factors is essential for managing cardiovascular health at the individual patient level (Levine et al., 2021). Psychophysiological factors not only modify CVD risk but can also independently predict adverse clinical outcomes (Pedersen et al., 2017). In support of this notion, we previously showed that the predominant α‐ and β‐adrenergic reactivity profiles are cross‐sectionally associated with increased cardiometabolic risk (Wentzel et al., 2025). Therefore, targeted psychophysiological interventions based on an individual's stress reactivity are needed. This requires both (i) a better understanding of neuroendocrine mechanisms associated with stress‐related CVDs and (ii) the identification of specific acute mental stress‐induced adrenergic–hemodynamic reactivity patterns.

A limitation of this approach is that acute mental stress testing is typically performed in a controlled laboratory environment rather than a clinical setting where patients receive medical care. This is largely due to the lack of specialized stress testing equipment (such as the Finometer) and standardized laboratory stressors (such as the Stroop‐CWC test), which are commonly used in cardiovascular reactivity studies. Therefore, exploring simpler and more accessible alternatives to identify an individual's predominant hemodynamic stress response pattern could be a promising avenue for better understanding the CVD risk associated with the different adrenergic‐hemodynamic reactivity profiles.

In this study, we identified distinct neuroendocrine signatures that may differentiate between predominant α‐adrenergic and β‐adrenergic responders based on urinary NE levels alone or in combination with serum ACTH and cortisol levels. The predominant α‐adrenergic reactivity profile appears to be characterized by elevated neuroendocrine markers reflecting both SAM (NE) and HPA (ACTH and cortisol) axis activity. In contrast, the predominant β‐adrenergic reactivity profile may be distinguished by elevated urinary NE levels exclusively. This suggests a potential model for distinguishing between adrenergic‐hemodynamic reactivity profiles and determining an individual's specific predominant hemodynamic reactivity response pattern using a urine sample (for NE) and a blood sample (for HPA markers), both of which could be obtained during a routine appointment. Such an approach enables personalized, targeted interventions that specifically address psychophysiological risk factors, promoting a precision medicine strategy for managing stress‐related CVDs rather than a generalized “one‐size‐fits‐all” approach (Pedersen et al., 2017). While both ACTH and cortisol are routinely measurable in fasting serum samples in most South African laboratories, these assays are not necessarily cost‐effective across all socioeconomic settings in South Africa (PathCare Laboratories, 2026). Together with restricted availability of free catecholamine assays (norepinephrine; epinephrine; dopamine) in these laboratories, their implementation into routine clinical practice for hemodynamic profiling is currently economically constrained. In addition, profile‐specific cut‐off values for hemodynamic phenotyping are yet to be established and validated against distinct cardiovascular outcomes. Nevertheless, our findings demonstrate the viability of following such a hemodynamic profiling approach and should be verified and validated in larger, high‐risk cohorts (e.g., individuals with hypertension, shift workers) and across diverse socioeconomic settings in South Africa. Validation against cardiovascular outcomes would support guidelines, similar to brain natriuretic peptide for heart failure (Kalsmith, 2009).

### 4.5 Strengths, limitations, and future directions

Our findings need to be interpreted within the study's strengths and limitations. The study was cross‐sectional, so we are unable to draw causal inferences from the observed associations. A longitudinal study design is recommended to confirm causal, mechanistic relationships between neuroendocrine markers and hemodynamic reactivity parameters in adrenergic responder groups. Although our study achieved the necessary statistical power and was limited by a small sample size, we obtained a homogeneous sample of teachers that is representative of the specific region (North West province) where the research was conducted. However, our findings cannot be generalized to the broader South African population.

A cohort of schoolteachers enabled us to investigate individuals that experience a similar level of sustained stress, such as adapting to changing curricula and disciplinary problems of school children whilst living in an urbanized environment. The participants in our study were recruited from the same demographic region in South Africa from a shared professional environment to ensure broadly comparable socioeconomic and educational backgrounds as well as similar access to healthcare. In our study, data on total household income and expenditure were not available, thus preventing us from distinguishing between single‐ and dual‐earner households or assessment of income allocation. Due to unique socio‐cultural responsibilities in South Africa, financial resources are often used to support family members within and outside their immediate households (Moore & Kelly, 2024), therefore, future studies may include structured questionnaires focused on socioeconomic information to capture these variations in household composition and income distribution more robustly.

While acute mental stress responses, as measured in the laboratory, remain stable over time (Hughes, 2013) and relatively robust across different types of stressors (Hamer et al., 2006; Hamer & Steptoe, 2012), future studies may investigate whether the observed patterns of these adrenergic responder groups hold true in more diverse populations of varying professions, ages, and ethnic groups. This study aimed to better understand stress reactivity from a physiological perspective and not from an angle focused on individual perceptions of stress. It would therefore be insightful in future studies to include data on self‐reported stress perception and appraisal questionnaires such as the Perceived Stress Scale (Cohen et al., 1983) or Trier Inventory for Chronic Stress (Schulz et al., 2004) to better understand the observed associations in individuals with high and low chronic stress based on their perception of stress and domain‐specific stressors they experience in daily life.

Predominant α‐ and β‐adrenergic reactivity response patterns were non‐invasively derived from peak hemodynamic reactivity, rather than from post‐stressor hemodynamic recovery. Importantly, reactivity and recovery represent two distinct physiological concepts that if conflated, could risk misclassification of adrenergic stress response patterns. Peak reactivity reflects maximal stressor exposure driven by rapid SNS outflow via α‐ and β‐adrenergic receptors (Obrist, 1981). Profiling reactivity allows the identification of predominant patterns (i.e., alpha: low CO/high TPR versus beta: high CO/low TPR) of extreme hemodynamic shifts from baseline, which could serve as prognostic markers for cardiometabolic risk (Wentzel et al., 2025). In contrast, recovery responses (measured 3–5 min post‐stressor) reflect rapid hemodynamic normalization via baroreflex buffering and parasympathetic rebound, not SNS peak (Mezzacappa et al., 2001). Recovery patterns may invert, for example, persistently elevated TPR could signal poor vascular recovery (α‐linked) whereas rapid decline in CO may suggest efficient myocardial reset (β‐influenced). Additionally, recovery probes resilience with slower DBP/TPR return tied to SNS hyperactivity or endothelial dysfunction. Yet this does not indicate peak reactivity during application of a stressor. Recovery assesses termination efficiency, often decoupled due to feedback loops like vagal reactivation (Haynes et al., 1991). As the aim of this study was to refine adrenergic response patterns, peak reactivity is required to characterize these patterns, not recovery. Accurate patterning therefore requires separate peak (stressor midpoint) and post‐peak (3 or 5 min) metrics to distinguish acute drive from adaptive resolution (Linden et al., 1997) and should be noted in future studies.

Although beyond the scope of the current study, it may be prudent to explore psychophysiological interventions tailored to each adrenergic reactivity phenotype, where predominant α‐adrenergic responders could possibly benefit from vascular‐related interventions which could improve faster post‐stress vascular recovery (Cahu Rodrigues et al., 2020), while predominant β‐adrenergic responders may benefit from more cardiac‐centered strategies which could enhance vagal HR control (Nolan et al., 2005) and preserve cardiac performance during persistent acute stress exposure. In addition, standardized clinical procedures were followed for measuring stress biomarkers through urinary and serum samples. Although urinary catecholamines are not considered gold‐standard biomarkers for sympathetic activity, their use provided a less invasive and more practical approach for basic clinical assessment of SAM‐biomarkers. Our study identifies unique neuroendocrine signatures which distinctly relate to each adrenergic reactivity profile and thus supports cortisol‐NE interplay. Yet the cross‐sectional nature of our study limits causal inferences on cortisol's permissive effects on NE functioning. We recommend that longitudinal studies tracking serial cortisol and catecholamine sampling, paired with hemodynamic reactivity, could elucidate dynamic cortisol‐NE interactions. Also, human microneurography offers direct sympathetic nerve assessment but is technically demanding; alternatives include PET/SPECT imaging of α‐adrenergic receptor occupancy or functional MRI for central NE signaling. Complementary in vitro receptor docking simulations and human adipocyte/lymphocyte assays could model cortisol's permissive effects on NE sensitivity, bridging to clinical trials stratifying interventions by adrenergic profiles. Future studies may also make use of clustering algorithms to delineate domain‐specific clusters of biological parameters in order to explore unique physiological patterns which could elucidate more intrinsic mechanisms and risks within each adrenergic responder group, yet this was beyond the aim of the current study. Finally, this study was carefully designed, conducted under controlled conditions, and implemented with measures to ensure optimal environmental stability.

## 5 CONCLUSION

The study is the first to report unique associations between neuroendocrine markers and predominant α‐ or β‐adrenergic reactivity profiles. Specifically, the predominant α‐adrenergic reactivity profile appears to be characterized by higher urinary NE and serum ACTH and cortisol levels, whereas the predominant β‐adrenergic reactivity profile may be identified based on elevated urinary NE levels alone. This suggests that HPA markers (ACTH and cortisol) may serve as key differentiators between these two adrenergic‐hemodynamic reactivity profiles. Additionally, neuroendocrine markers associated differently in both profiles. These distinct neuroendocrine signatures could facilitate the accurate identification of acute adrenergic‐hemodynamic reactivity profiles, informing individual risk assessment and targeted treatment strategies based on a patient's adrenergic‐hemodynamic reactivity profile.

## AUTHOR CONTRIBUTIONS

**Dewald Naudé:** Conceptualization, formal analysis, visualization, writing—original draft, writing—review and editing. **Wayne Smith:** Conceptualization, data curation, investigation, methodology, supervision, writing—original draft, writing—review and editing. **Roland von Känel:** Data curation, supervision, writing—review and editing. **Annemarie Wentzel:** Conceptualization, data curation, formal analysis, funding acquisition, investigation, methodology, project administration, supervision, writing—original draft, writing—review and editing.

## FUNDING INFORMATION

This work was supported by North‐West University and North‐West Education Department South Africa; Medical Research Council and National Research Foundation South Africa; ROCHE Diagnostics South Africa; Heart and Stroke Foundation South Africa; and the Metabolic Syndrome Institute, France. Any opinions, findings, and conclusions or recommendations expressed in this material are those of the author(s), and therefore funding bodies do not accept any liability in regard thereto.

## CONFLICT OF INTEREST STATEMENT

The authors have nothing to report.

## DISCLAIMERS

Any opinion, findings and conclusions or recommendations expressed in this material are those of the authors; therefore, funders do not accept any liability regarding this study. All co‐authors approve the statements made on the cover letter attached to the submission of this original research article.

## Supporting information


Data S1.

## Data Availability

All enquiries regarding data availability can be made upon reasonable request to the corresponding author, Annemarie Wentzel.
