# Supplementary material for: Resting neuroendocrine markers in relation to acute mental stress‐induced adrenergic reactivity profiles in adults: The SABPA study
Source: Physiol Rep. 2026 Mar 16;14(6):e70809. doi: 10.14814/phy2.70809 (PMC13097463; doi:10.14814/phy2.70809)
Supplement: Supplementary file 1 — Data S1. [file PHY2-14-e70809-s001.docx]

| **Table S1:** Spearman rank correlations between cardiac output reactivity (∆%CO) with main independent variables and confounders in acute mental stress-induced adrenergic-haemodynamic reactivity profiles (N=375) | | | | | | | | | | |
| --- | --- | --- | --- | --- | --- | --- | --- | --- | --- | --- |
|  | | **Dependent variable: Cardiac output reactivity (∆%CO)** | | | | | | | | |
|  | | Total population  (N=375) | | | Predominant α-adrenergic  reactivity profile (n=49) | | Mixed-α/β-adrenergic  reactivity profile (n=257) | | Predominant β-adrenergic  reactivity profile (n=69) | |
| Age, years | | **r = -0.20; p<0.001** | | | r = -0.13; p=0.38 | | r = -0.088; p=0.16 | | r = -0.074; p=0.55 | |
| Sex | | r = -0.026; p=0.61 | | | r = 0.038; p=0.80 | | r = -0.002; p=0.98 | | r = -0.032; p=0.79 | |
| Ethnicity | | **r = 0.26; p<0.001** | | | r = 0.090; p=0.54 | | **r = 0.18; p=0.004** | | r = 0.061; p=0.62 | |
| ACTH, pg/mL | | **r = -0.12; p=0.022** | | | r = -0.023; p=0.87 | | r = -0.098; p=0.12 | | r = -0.080; p=0.52 | |
| Cortisol, nmol/L | | r = -0.012; p=0.81 | | | r = 0.079; p=0.59 | | r = -0.060; p=0.34 | | r = 0.18; p=0.15 | |
| u-NE/Cr, nmol/mmol | | r = -0.034; p=0.51 | | | r = 0.17; p=0.24 | | r = 0.019; p=0.77 | | r = -0.23; p=0.059 | |
| u-EPI/Cr, nmol/mmol | | r = 0.045; p=0.39 | | | r = 0.23; p=0.12 | | r = 0.094; p=0.13 | | r = -0.10; p=0.40 | |
| Body mass index, kg/m^2^ | | **r = -0.27; p<0.001** | | | r = -0.26; p=0.073 | | **r = -0.19; p=0.002** | | **r = -0.32; p=0.007** | |
| Waist circumference, cm | | **r = -0.23; p<0.001** | | | **r = -0.32; p=0.025** | | **r = -0.15; p=0.016** | | **r = -0.31; p=0.010** | |
| 24-hour ABPM SBP, mmHg | | **r = -0.32; p<0.001** | | | r = -0.062; p=0.67 | | **r = -0.18; p=0.005** | | **r = -0.29; p=0.018** | |
| 24-hour ABPM DBP, mmHg | | **r = -0.24; p<0.001** | | | r = -0.014; p=0.92 | | r = -0.11; p=0.091 | | **r = -0.36; p=0.002** | |
| 24-hour ABPM MAP, mmHg | | **r = -0.28; p<0.001** | | | r = -0.033; p=0.82 | | **r = -0.14; p=0.028** | | **r = -0.36; p=0.003** | |
| Hypertensive status | | **r = -0.29; p<0.001** | | | r = -0.066; p=0.65 | | **r = -0.18; p=0.003** | | r = -0.17; p=0.15 | |
| Glycated haemoglobin, % | | **r = -0.26; p<0.001** | | | r = -0.14; p=0.33 | | **r = -0.16; p=0.011** | | r = -0.027; p=0.83 | |
| Glucose, mmol/L | | r = -0.072; p=0.16 | | | r = -0.034; p=0.82 | | r = 0.007; p=0.91 | | r = 0.011; p=0.93 | |
| Insulin, μU/mL | | **r = -0.27; p<0.001** | | | **r = -0.42; p=0.003** | | **r = -0.18; p=0.003** | | **r = -0.33; p=0.005** | |
| HOMA-IR | | **r = -0.26; p<0.001** | | | **r = -0.32; p=0.025** | | **r = -0.16; p=0.010** | | **r = -0.31; p=0.010** | |
| Abnormal glucose tolerance | | **r = -0.21; p<0.001** | | | **r = -0.31; p=0.032** | | r = -0.14; p=0.031 | | r = 0.027; p=0.82 | |
| C-reactive protein, mg/L | | **r = -0.27; p<0.001** | | | r = -0.024; p=0.87 | | **r = -0.19; p=0.002** | | **r = -0.38; p=0.001** | |
| TNF-α, pg/mL | | **r = -0.11; p=0.027** | | | r = 0.032; p=0.83 | | r = -0.050; p=0.43 | | r = -0.043; p=0.73 | |
| Interleukin-6, pg/mL | | **r = -0.21; p<0.001** | | | r = -0.030; p=0.84 | | **r = -0.20; p=0.002** | | **r = -0.30; p=0.012** | |
| Total cholesterol, mmol/L | | r = 0.010; p=0.85 | | | r = 0.12; p=0.40 | | r = 0.029; p=0.64 | | **r = -0.27; p=0.026** | |
| HDL-cholesterol, mmol/L | | **r = 0.15; p=0.004** | | | r = 0.26; p=0.075 | | r = 0.11; p=0.093 | | **r = 0.28; p=0.019** | |
| LDL-cholesterol, mmol/L | | r = -0.014; p=0.79 | | | r = 0.068; p=0.64 | | r = 0.001; p=0.99 | | **r = -0.35; p=0.003** | |
| Triglycerides, mmol/L | | **r = -0.18; p<0.001** | | | r = -0.24; p=0.10 | | r = -0.049; p=0.43 | | **r = -0.34; p=0.004** | |
| Cholesterol-to-HDL-C ratio | | **r = -0.13; p = 0.015** | | | r = -0.17; p = 0.25 | | r = -0.065; p=0.30 | | **r = -0.46; p<0.001** | |
| eGFR, mL/min/1.73 m^2^ | | r = 0.098; p=0.058 | | | **r = 0.36; p=0.012** | | r = 0.066; p=0.29 | | r = -0.054; p=0.66 | |
| Self-reported alcohol | | r = -0.010; p=0.85 | | | r = -0.18; p=0.46 | | r = -0.09; p=0.19 | | r = -0.19; p=0.73 | |
| Self-reported smoking | | r = -0.034; p=0.51 | | | r = 0.23; p=0.90 | | r = -0.08; p=0.22 | | r = 0.12; p=0.65 | |
| Regression coefficients and p-values were obtained with Spearman rank correlation analyses. Bold values denote statistical significance (p<0.050). Abbreviations: ABPM, ambulatory blood pressure monitoring; ACTH, adrenocorticotropic hormone; DBP, diastolic blood pressure; HOMA-IR, homeostatic model assessment for insulin resistance; MAP, mean arterial pressure; SBP, systolic blood pressure; TNF-α, tumour necrosis factor-alpha; u-NE/Cr, urinary norepinephrine-to-creatinine ratio, u-EPI/Cr, urinary epinephrine-to-creatinine ratio | | | | | | | | | | |
| **Table S2:** Spearman rank correlations between Windkessel arterial compliance reactivity (∆%Cwk) with main independent variables and confounders in acute mental stress-induced adrenergic-haemodynamic reactivity profiles (N=375) | | | | | | | | | | |
|  | | **Dependent variable: Windkessel arterial compliance reactivity (∆%Cwk)** | | | | | | | | |
|  | | Total population  (N=375) | | | Predominant α-adrenergic  reactivity profile (n=49) | | Mixed-α/β-adrenergic  reactivity profile (n=257) | | Predominant β-adrenergic  reactivity profile (n=69) | |
| Age, years | | **r = -0.18; p<0.001** | | | r = -0.068; p=0.64 | | **r = -0.13; p=0.034** | | r = 0.24; p=0.051 | |
| Sex | | r = -0.059; p=0.26 | | | r = -0.040; p=0.78 | | r = -0.079; p=0.21 | | r = 0.057; p=0.64 | |
| Ethnicity | | **r = 0.16; p=0.002** | | | r = 0.21; p=0.15 | | r = 0.012; p=0.85 | | r = 0.10; p=0.42 | |
| ACTH, pg/mL | | r = -0.060; p=0.25 | | | r = 0.021; p=0.89 | | r = -0.030; p=0.64 | | r = 0.058; p=0.63 | |
| Cortisol, nmol/L | | r = -0.048; p=0.36 | | | r = -0.13; p=0.39 | | r = 0.019; p=0.76 | | r = -0.17; p=0.17 | |
| u-NE/Cr, nmol/mmol | | r = 0.055; p=0.29 | | | **r = 0.33; p=0.019** | | r = 0.095; p=0.13 | | r = 0.013; p=0.92 | |
| u-EPI/Cr, nmol/mmol | | r = -0.093; p=0.071 | | | r = 0.054; p=0.71 | | r = -0.10; p=0.11 | | r = 0.003; p=0.98 | |
| Body mass index, kg/m^2^ | | r = 0.015; p=0.78 | | | r = -0.053; p=0.72 | | r = 0.11; p=0.086 | | **r = 0.29; p=0.017** | |
| Waist circumference, cm | | r = -0.040; p=0.44 | | | r = -0.16; p=0.27 | | r = 0.014; p=0.83 | | r = 0.18; p=0.13 | |
| 24-hour ABPM SBP, mmHg | | **r = -0.16; p=0.002** | | | **r = -0.36; p=0.011** | | r = 0.025; p=0.69 | | r = 0.10; p=0.41 | |
| 24-hour ABPM DBP, mmHg | | **r = -0.14; p=0.006** | | | r = -0.19; p=0.19 | | r = -0.019; p=0.76 | | r = 0.050; p=0.68 | |
| 24-hour ABPM MAP, mmHg | | **r = -0.16; p=0.003** | | | **r = -0.29; p=0.043** | | r = 0.002; p=0.98 | | r = 0.073; p=0.55 | |
| Hypertensive status | | **r = -0.12; p=0.022** | | | r = -0.17; p=0.25 | | r = 0.016; p=0.79 | | r = 0.20; p=0.098 | |
| Glycated hemoglobin, % | | **r = -0.10; p=0.050** | | | r = -0.031; p=0.83 | | r = 0.009; p=0.89 | | **r = 0.29; p=0.018** | |
| Glucose, mmol/L | | **r = -0.12; p=0.020** | | | r = -0.036; p=0.81 | | r = -0.067; p=0.29 | | r = 0.072; p=0.56 | |
| Insulin, μU/mL | | r = -0.036; p=0.48 | | | r = -0.088; p=0.55 | | r = 0.020; p=0.76 | | **r = 0.27; p=0.028** | |
| HOMA-IR | | r = -0.074; p=0.15 | | | r = -0.074; p=0.61 | | r = -0.013; p=0.84 | | r = 0.21; p=0.087 | |
| Abnormal glucose tolerance | | r = -0.088; p=0.091 | | | r = -0.010; p=0.95 | | r = 0.019; p=0.76 | | r = 0.17; p=0.16 | |
| C-reactive protein, mg/L | | r = -0.041; p=0.43 | | | r = 0.084; p=0.57 | | r = 0.047; p=0.45 | | r = 0.093; p=0.45 | |
| TNF-α, pg/mL | | r = -0.029; p=0.57 | | | r = -0.12; p=0.42 | | r = 0.033; p=0.60 | | r = 0.19; p=0.12 | |
| Interleukin-6, pg/mL | | r = 0.005; p=0.93 | | | r = -0.13; p=0.40 | | r = 0.071; p=0.25 | | r = 0.11; p=0.35 | |
| Total cholesterol, mmol/L | | r = 0.029; p=0.57 | | | r = 0.10; p=0.49 | | r = -0.020; p=0.75 | | r = 0.10; p=0.39 | |
| HDL-cholesterol, mmol/L | | r = -0.021; p=0.69 | | | r = 0.14; p=0.34 | | r = -0.085; p=0.18 | | r = -0.067; p=0.58 | |
| LDL-cholesterol, mmol/L | | r = 0.056; p=0.28 | | | r = 0.079; p=0.59 | | r = 0.004; p=0.95 | | r = 0.16; p=0.19 | |
| Triglycerides, mmol/L | | **r = -0.11; p=0.038** | | | r = -0.11; p=0.47 | | r = -0.031; p=0.62 | | r = -0.001; p=0.99 | |
| Cholesterol-to-HDL-C ratio | | r = 0.040; p=0.44 | | | r = -0.052; p=0.72 | | r = 0.055; p=0.38 | | r = 0.17; p=0.16 | |
| eGFR, mL/min/1.73 m^2^ | | r = 0.082; p=0.12 | | | r = 0.16; p=0.27 | | r = 0.10; p=0.11 | | **r = -0.31; p=0.010** | |
| Self-reported alcohol | | **r = -0.19; p=0.040** | | | r = -0.12; p=0.70 | | r = -0.10; p=0.25 | | r = 0.06; p=0.77 | |
| Self-reported smoking | | r = -0.23; p=0.24 | | | r = 0.23; p=0.099 | | r = -0.08; p=0.12 | | r = -0.12; p=0.34 | |
| Regression coefficients and p-values were obtained with Spearman rank correlation analyses. Bold values denote statistical significance (p<0.050). Abbreviations: ABPM, ambulatory blood pressure monitoring; ACTH, adrenocorticotropic hormone; DBP, diastolic blood pressure; HOMA-IR, homeostatic model assessment for insulin resistance; MAP, mean arterial pressure; SBP, systolic blood pressure; TNF-α, tumour necrosis factor-alpha; u-NE/Cr, urinary norepinephrine-to-creatinine ratio, u-EPI/Cr, urinary epinephrine-to-creatinine ratio | | | | | | | | | | |
| **Table S3:** Backward multiple regression analyses of resting neuroendocrine markers and stroke volume reactivity (∆%SV) stratified according to acute mental stress-induced adrenergic-haemodynamic reactivity profiles (N=375) | | | | | | | | | | |
|  | Predominant α-adrenergic  reactivity profile (n=49) | | | Mixed-α/β-adrenergic  reactivity profile (n=257) | | | | Predominant β-adrenergic  reactivity profile (n=69) | | |
| **Dependent variable**: Stroke volume reactivity (∆%SV) | | | | | | | | | | |
|  | **ß (± 95% CI)** | | ***p*** | **ß (± 95% CI)** | | ***p*** | | **ß (± 95% CI)** | | ***P*** |
| **Adj R^2^** | 0.41 | | | <0.10 | | | | 0.36 | | |
| **u-NE/Cr** | -1.65 (-2.14; -1.10) | | **0.003** | 2.16 (0.61; 3.71) | | **0.007** | | 5.23 (3.07; 7.21) | | **0.019** |
| Age | NS | | NS | -0.14 (-0.28; 0.007) | | 0.062 | | NS | | NS |
| Ethnicity | NS | | NS | NS | | NS | | NS | | NS |
| Waist circumference | NS | | NS | 0.14 (0.048; 0.23) | | **0.003** | | NS | | NS |
| C-reactive protein | NS | | NS | -1.48 (-2.64; 1.22) | | 0.098 | | NS | | NS |
| eGFR | 0.21 (0.026; 0.40) | | **0.027** | NS | | NS | | NS | | NS |
| Abnl-GT | NS | | NS | NS | | NS | | 8.58 (5.18; 12.7) | | **0.004** |
| **Adj R^2^** | 0.19 | | | 0.10 | | | | 0.20 | | |
| **u-EPI/Cr** | -0.94 (-1.27; -0.35) | | **0.048** | 2.18 (0.55; 3.81) | | **0.009** | | 5.54 (3.56; 8.32) | | **0.012** |
| Age | NS | | NS | -0.14 (-0.29; 0.004) | | 0.056 | | NS | | NS |
| Ethnicity | NS | | NS | 3.48 (0.61; 6.35) | | **0.018** | | NS | | NS |
| Waist circumference | NS | | NS | 0.15 (0.060; 0.24) | | **0.001** | | NS | | NS |
| C-reactive protein | NS | | NS | -1.47 (-2.83; 0.01) | | 0.054 | | NS | | NS |
| eGFR | 0.21 (-0.03; 0.40) | | 0.059 | NS | | NS | | NS | | NS |
| Abnl-GT | NS | | NS | NS | | NS | | 6.32 (3.69; 9.04) | | **0.013** |
| **Adj R^2^** | 0.40 | | | 0.10 | | | | <0.010 | | |
| **ACTH** | -1.03 (-1.55; -0.74) | | **0.002** | -2.67 (-5.06; -0.28) | | **0.029** | | NS | | NS |
| Waist circumference | NS | | NS | 0.21 (0.11; 0.31) | | **<0.001** | | NS | | NS |
| 24-hour ABPM MAP | NS | | NS | -0.16 (-0.29; -0.018) | | **0.027** | | NS | | NS |
| C-reactive protein | NS | | NS | -2.13 (-2.49; 0.08) | | 0.051 | | NS | | NS |
| eGFR | 0.21 (0.026; 0.40) | | **0.027** | NS | | NS | | NS | | NS |
| Abnl-GT | NS | | NS | NS | | NS | | 6.75 (3.87; 9.16) | | **0.007** |
| **Adj R^2^** | 0.31 | | | 0.10 | | | | <0.10 | | |
| **Cortisol** | -5.28 (-8.91; -2.15) | | **0.008** | NS | | NS | | NS | | NS |
| Ethnicity | NS | | NS | 3.22 (0.35; 6.08) | | **0.028** | | NS | | NS |
| Waist circumference | NS | | NS | 0.17 (0.073; 0.27) | | **<0.001** | | NS | | NS |
| Abnl-GT | NS | | NS | -2.79 (-5.81; 0.23) | | 0.070 | | 8.36 (2.98; 13.4) | | **0.012** |
| C-reactive protein | NS | | NS | -0.59 (-1.72; -0.18) | | **0.036** | | NS | | NS |
| eGFR | 0.19 (0.016; 0.37) | | **0.033** | NS | | NS | | NS | | NS |
| All models were adjusted for age, sex, ethnicity, waist circumference, 24-hour ambulatory mean arterial pressure (ABPM MAP), abnormal glucose tolerance (Abnl-GT), self-reported smoking and self-reported alcohol use. For models specific to α-adrenergic responders, estimated glomerular filtration rate (eGFR) was added. For models specific to mixed-α/β-adrenergic responders, C-reactive protein and cholesterol-to-HDL ratio were added. For models specific to β-adrenergic responders, interleukin-6 was added. Abbreviations: ACTH, adrenocorticotropic hormone; CI, confidence interval; NS, not significant; u-EPI/Cr, urinary epinephrine-to-creatinine ratio; u-NE/Cr, urinary norepinephrine-to-creatinine ratio. Bold values denote statistical significance (*p*<0.050). | | | | | | | | | | |

| **Table S4:** Backward multiple regression analyses of resting neuroendocrine markers and total peripheral resistance reactivity (∆%TPR) stratified according to acute mental stress-induced adrenergic-haemodynamic reactivity profiles (N=375) | | | | | | |
| --- | --- | --- | --- | --- | --- | --- |
|  | Predominant α-adrenergic  reactivity profile (n=49) | | Mixed-α/β-adrenergic  reactivity profile (n=257) | | Predominant β-adrenergic  reactivity profile (n=69) | |
| **Dependent variable:** Total peripheral resistance reactivity (∆%TPR) | | | | | | |
|  | **ß (± 95% CI)** | ***p*** | **ß (± 95% CI)** | ***p*** | **ß (± 95% CI)** | ***P*** |
| **Adj R^2^** | 0.25 | | <0.10 | | 0.29 | |
| **u-NE/Cr** | 0.78 (0.35; 1.02) | **0.029** | NS | NS | -1.36 (-1.69; -0.94) | **0.033** |
| 24-hour ABPM MAP | NS | NS | NS | NS | 0.68 (0.32; 0.97) | **0.028** |
| **Adj R^2^** | 0.12 | | <0.10 | | 0.19 | |
| **u-EPI/Cr** | 0.22 (-0.09; 0.56) | 0.052 | NS | NS | -0.32 (-0.65; -0.07) | 0.075 |
| 24-hour ABPM MAP | NS | NS | NS | NS | 0.39 (0.07; 0.71) | **0.011** |
| **Adj R^2^** | 0.23 | | <0.10 | | 0.11 | |
| **ACTH** | 8.69 (2.65; 14.0) | **0.007** | NS | NS | NS | NS |
| Waist circumference | 2.48 (0.72; 4.58) | **0.021** | 0.79 (0.36; 1.29) | **0.019** | 0.35 (0.15; 0.63) | 0.069 |
| 24-hour ABPM MAP | NS | NS | NS | NS | 0.81 (0.55; 1.12) | **0.006** |
| **Adj R^2^** | 0.36 | | <0.10 | | 0.13 | |
| **Cortisol** | 2.12 (1.63; 2.78) | **0.043** | NS | NS | -0.23 (-0.14; -0.47) | **0.018** |
| 24-hour ABPM MAP | NS | NS | NS | NS | 0.68 (0.32; 1.09) | **0.009** |
| All models were adjusted for age, sex, ethnicity, waist circumference, 24-hour ambulatory mean arterial pressure (ABPM MAP), abnormal glucose tolerance, self-reported smoking and self-reported alcohol use. For models specific to α-adrenergic responders, estimated glomerular filtration rate was added. For models specific to mixed-α/β-adrenergic responders, C-reactive protein and cholesterol-to-HDL ratio were added. For models specific to β-adrenergic responders, interleukin-6 was added. Abbreviations: ACTH, adrenocorticotropic hormone; CI, confidence interval; NS, not significant; u-EPI/Cr, urinary epinephrine-to-creatinine ratio; u-NE/Cr, urinary norepinephrine-to-creatinine ratio. Bold values denote statistical significance (*p*<0.050). | | | | | | |
